# Emerging role of LETM1/GRP78 axis in lung cancer

**DOI:** 10.1038/s41419-022-04993-5

**Published:** 2022-06-10

**Authors:** Quangdon Tran, Hyunji Lee, Jae Hun Jung, Seung-Hee Chang, Robin Shrestha, Gyeyeong Kong, Jisoo Park, Seon-Hwan Kim, Kyu-Sang Park, Hyun-Woo Rhee, Jeanho Yun, Myung-Haing Cho, Kwang Pyo Kim, Jongsun Park

**Affiliations:** 1 Department of Pharmacology, College of Medicine, Chungnam National University, Daejeon, 35015 South Korea; 2 Department of Medical Science, Metabolic Syndrome and Cell Signaling Laboratory, Institute for Cancer Research, College of Medicine, Chungnam National University, Daejeon, 35015 South Korea; 3 Molecular Biology Laboratory, Department of Medical Laboratories, Hai Phong International Hospital, Hai Phong City, #18000 Vietnam; 4 Department of Applied Chemistry, College of Applied Sciences, Kyunghee University, Yongin, 17104 South Korea; 5 Laboratory of Toxicology, College of Veterinary Medicine Seoul National University, Seoul, 08826 South Korea; 6 Department of Life Science, Hyehwa Liberal Arts College, Daejeon University, Daejeon, 34520 South Korea; 7 Department of Neurosurgery, Institute for Cancer Research, College of Medicine, Chungnam National University, Daejeon, 35015 South Korea; 8 Department of Physiology and Institute of Lifestyle Medicine, Yonsei University Wonju College of Medicine, Wonju, 26427 Korea; 9 Department of Chemistry, Ulsan National Institute of Science and Technology, Ulsan, 44919 Korea; 10 Mitochondria Hub Regulation Center, College of Medicine, Dong-A University, Busan, 49201 South Korea; 11 RNABIO, Seongnam, Gyeonggi-do 13201 South Korea

**Keywords:** Non-small-cell lung cancer, Mitophagy

## Abstract

The selective autophagy of damaged mitochondria is called mitophagy. Mitochondrial dysfunction, mitophagy, and apoptosis have been suggested to be interrelated in various human lung carcinomas. Leucine zipper EF-hand-containing transmembrane protein-1 (LETM1) was cloned in an attempt to identify candidate genes for Wolf–Hirschhorn syndrome. LETM1 plays a role in mitochondrial morphology, ion homeostasis, and cell viability. LETM1 has also been shown to be overexpressed in different human cancer tissues, including lung cancer. In the current study, we have provided clear evidence that LETM1 acts as an anchoring protein for the mitochondria-associated ER membrane (MAM). Fragmented mitochondria have been found in lung cancer cells with LETM1 overexpression. In addition, a reduction of mitochondrial membrane potential and significant accumulation of microtubule-associated protein 1 A/1B-light chain 3 punctate, which localizes with Red-Mito, was found in LETM1-overexpressed cells, suggesting that mitophagy is upregulated in these cells. Interestingly, glucose-regulated protein 78 kDa (GRP78; an ER chaperon protein) and glucose-regulated protein 75 kDa (GRP75) were posited to interact with LETM1 in the immunoprecipitated LETM1 of H460 cells. This interaction was enhanced in cells treated with carbonyl cyanide m-chlorophenylhydrazone, a chemical mitophagy inducer. Treatment of cells with honokiol (a GRP78 inhibitor) blocked LETM1-mediated mitophagy, and CRISPR/Cas9-mediated GRP75 knockout inhibited LETM1-induced autophagy. Thus, GRP78 interacts with LETM1. Taken together, these observations support the notion that the complex formation of LETM1/GRP75/GRP78 might be an important step in MAM formation and mitophagy, thus regulating mitochondrial quality control in lung cancer.

## Introduction

Mitochondria are key regulators of many cellular processes, involving in a range of pathophysiologic conditions, including cancer and inflammation [1]. Based on the Warburg theory, which posits that cancer cells rely on glycolytic ATP production despite the presence of oxygen, mitochondrial DNA mutations and physiological dysfunctions have been extensively studied to elucidate the mechanisms underlying the malfunction of respiratory chains, which renders the cells dependent on glycolysis to supply their ATP [2]. Even though it has yet to be clarified whether mitochondrial defects are a cause or a consequence of cancer development, mitochondrial dysfunction has been recognized as a common event in various cancers [1]. Quality control of mitochondria is tightly regulated to avoid destructive effects of malfunctioning mitochondria, and to sustain the health of cells [3, 4], where mitochondria play pivotal roles in cellular ATP production, apoptosis, and other normal cellular activities [5–7].

Mitophagy is suggested to be a major mechanism in the quality control of mitochondria, which involves a selective autophagic process for damaged or unnecessary mitochondria [8]. Autophagy refers to the process of lysosomal-mediated degradation of intracellular contents for subsequent reutilization [9]. Regarding cancer initiation, autophagy performs an anti-tumorigenic role by clearing dysfunctional mitochondria and abnormal proteins [10, 11]. However, in the case of cancer progression, tumor cells overcome metabolic stress by using autophagy to gain nutrients from recycled organelles and proteins [12]. In addition, the depletion of autophagy proteins leads to DNA damage and genetic instability, resulting in tumorigenesis [12]. Lemasters and colleagues demonstrated that treatment of rat hepatocytes with glucagon in the absence of serum led to the depolarization of mitochondria and acidic lysosomes, which is indicative of mitochondria-specific autophagy [13]. Accumulating evidence suggests that dysfunction and morphological changes in mitochondria are responsible for mitophagy induction [14–16].

Leucine zipper EF-hand-containing transmembrane protein 1 (LETM1) was initially identified as a gene deletion in Wolf–Hirschhorn syndrome (WHS) [17] and appeared to control mitochondrial morphology, ion homeostasis, and cell viability [18–21]. In addition, LETM1 was shown to be overexpressed in different human cancers, including lung cancer (PMID: 31500591, 2024095) [19]. Consistent with the observation of LETM1-mediated optic atrophy 1 (OPA1) regulation [18], a reduction of OPA1 has been observed during mitophagy [16], suggesting a possible role of LETM1 in the induction of the mitophagic process. In the current study, we demonstrated a molercular role of LETM1 complex which involve in the mitochondria-ER association and facilitated the miochondrial regulation in lung cancer cells.

## Materials and methods

### Antibodies and reagents

Anti-LETM1 antibody was purchased from Abnova (Cat. H00003954-M03, clone 6F7, Taiwan). Anti-LC3B (Cat. L7543) and anti-Actin (Cat. A2228) antibodies were purchased from Sigma-Aldrich (St. Louis, USA). Anti-GRP78 (Cat. 3177), anti-tubulin (Cat. 2128 S) and anti-PINK1 (Cat. 6946) were obtained from Cell Signaling (Massachusetts, USA). Anti-GRP75 (Cat. sc-133137) and anti-Tom40 (Cat. sc-365467) were from Santacruz Biotech (Dallas, Texas, USA). Horseradish peroxidase-conjugated anti-mouse IgG or anti-rabbit IgG secondary antibodies were purchased from Komabiotech (Seoul, Korea). Carbonyl cyanide m-chlorophenyl hydrazine (CCCP) (215911), 3-methyladenine (3-MA) (M9281), chloroquine (CQ) (C6628) and honokiol (HNK) (H4914) were from Sigma-Aldrich (St. Louis, USA). Wortmannin (WM) (681676) was from Calbiochem (San Diego, USA).

### Expression vectors

Adenoviral expression vector for wild type LETM1 was prepared as the described previously [19]. pDsRed-Mito vector was purchased from Clontech (California, USA). Crispr/Cas9-GRP75 plasmid was from Santacruz Biotech (California, USA).

### Cell culture and stimulation

H460 cells were maintained in medium (RPMI) supplemented with 10% FBS (Life Technologies, Grand Island, USA), 25 mM HEPES (Healthcare life sciences, Utah, USA) and 1% Antibiotics-Antimycotics (Life Technologies, Grand Island, USA). A549 cells were maintained in medium (DMEM) supplemented with 10% FBS, 1% Antibiotics-Antimycotics. These cells were transiently transfected using Lipofectamine (Invitrogen, California, USA) or jet-PEI (Polyplus, New York, USA) reagents following the instructions provided by manufacturers. Adenovirus (Ad)-LacZ and Ad-LETM1 were infected for 24 h. These cells were treated with 30 μM CCCP for 8 h, 10 μM CQ for 6 h, 1 μM WM for 2 h, 25 μM HNK for 8 h or 10 μM 3-MA for 2 h. H460 cells were transiently transfected with short interference NC RNA (si-NC), si-LETM1 using Lipofectamine (Invitrogen, California, USA) reagents following the instructions provided by manufacturers.

### Immunoblot analysis

The immunoblot analysis was performed as the described previously [22, 23]. After the completion of experimental conditions, cells were placed on ice and extracted with lysis buffer containing 50 mM Tris-HCl, pH 7.5, 1% v/v Nonidet P-40, 120 mM NaCl, 25 mM sodium fluoride, 40 mM β-glycerol phosphate, 0.1 mM sodium orthovanadate, 1 mM phenylmethylsulfonyl fluoride, 1 mM benzamidine, and 2 μM microcystin-LR. Lysates were centrifuged for 15 min at x 12,000 g. The cell extracts were resolved by 15, 12 and 10 % SDS-PAGE, and transferred to Immobilon-P membranes (Millipore, Darmstadt, Germany). The filters were blocked for 1 hr in 1× tri-buffered saline buffer (TBS; 140 mM NaCl, 2.7 mM KCl, 250 mM Tris-HCl, pH 7.4), containing 5 % skimmed milk and 0.2 % Tween-20, followed by an overnight incubation with the primary antibodies diluted 1000 folds (1:1000) at 4 °C. The secondary antibody was horseradish peroxidase-conjugated anti-mouse IgG or anti-rabbit IgG (Komabiotech, Seoul, Korea), diluted 5000-fold (1:5000) in the blocking buffer. The detection of protein expression was visualized by enhanced chemiluminescence, according to the manufacturer’s instructions (Healthcare life sciences, Utah, USA).

### Immunoprecipitation assay

H460 cells were infected with Ad-LacZ and Ad-LETM1 for 24 hr, then placed on ice and extracted with lysis buffer containing 50 mM Tris-HCl (pH 7.5), 0.5% v/v Nonidet P-40, 250 mM NaCl, 3 mM EDTA (pH 8.0), 3 mM EGTA (pH 8.0), 40 mM h-glycerol phosphate, 0.1 mM sodium orthovanadate, 1 mM phenylmethylsulfonyl fluoride, 1 mM benzamidine, and 2 μM of microcystin-LR. LETM1 protein was immunoprecipitated from 1000 μg of cell-free extracts with normal IgG or LETM1 antibody. The immune complexes were washed three times, separated on SDS-PAGE and analyzed by immunoblotting with the corresponding antibodies.

### Proteomic analysis of LETM1 immunoprecipitates

H460 cells were infected with Ad-LacZ and Ad-LETM1 for 24 h, followed by immunoprecipitation with anti-LETM1 antibody. Samples were further analyzed by SDS-PAGE. The resultant gel lanes containing LETM1 were reduced, alkylated and digested with trypsin. The digested peptides were analyzed using Q-Exactive orbitrap hybrid mass spectrometer coupled with an EASY-nLC 1000 system (Thermo Fisher Scientific). All tandem mass spectrometry (MS/MS) spectra were acquired in a data-dependent mode for 8 most abundant peaks from the full mass spectrometry (MS) scan (m/z; 300–2000) with 27% normalized collision energy. Dynamic exclusion duration was 15 s and isolation window was set to 2.0 m/z. The MS/MS scans were acquired at a resolution of 17,500 (at m/z 200) with an automated gain control target value of 5.0 × 10^4^ and a maximum ion injection of 60 ms to get high quality of MS/MS spectrum. The acquired MS/MS spectra were searched against Uniprot Human database with the Sequest algorithm in Proteome Discoverer 1.3 (Thermo Fisher Scientific). Peptide spectra matched (PSM) number from identified proteins in each data set (Ad-LacZ and Ad-LETM1) were compared and significantly upregulated proteins were selected as a major putative interaction partners.

### Mitochondrial morphology analysis with confocal microscopy

H460 cells were grown on glass coverslips until they were 50–70% confluent, followed by transfection of cells with pDsRed-Mito constructs of GFP-LC3 constructs by using Lipofectamine or jet PEI reagents, followed by infection with the combination of proper adenovirus (Ad-LETM1 or Ad-LacZ). After 24 h, the cells were fixed in 4% paraformaldehyde at room temperature for 10 min and permeabilized in 0.2% Triton X100 for 15 min at room temperature. Then the coverslips were mounted with Vectashield (Vector Laboratories, Burlingame, CA) and visualized using a Zeiss confocal microscope.

### Subcellular structure analysis with transmission electron microscopy

H460 cells were infected with Ad-LacZ and Ad-LETM1 for 24 h and treated with CCCP for 8 h. Then the cells were fixed in a solution of 2.5% glutaraldehyde with 1% Osmium Oxide 4 (OsO_4_) buffer for 1 h and dehydrated with ethanol at 4 °C. Then cells were infiltrated in a 1:1 mixture of propylene oxide and Epon, and finally embedded in Epon by polymerization at 60 °C for 48 h. Ultrastructural analyses were performed on a SPIRIT G2 electron microscope.

### Measurement of mitochondrial membrane potential

H460 cells were infected with Ad-LacZ and Ad-LETM1 for 24 h. Then cells were collected and stained with JC-1 dye (Molecular probes, Invitrogen, Eugene, USA) for 30 min. The cells were treated with 30 μM CCCP for 10 min as a positive control to induce mitochondrial depolarization before staining with the dye. Finally, the cells were analyzed by FACS Canto system (Becton Dickinson, Oxford, UK).

### Measurement of mitochondrial matrix calcium

H460 cells were grown on coverslips until they were 50–70% confluent and then transfected with mitochondria-targeted ratio-pericam (mtRP) plasmid [24] for 24 h followed by infection with Ad-LacZ or Ad-LETM1 for 24 h. Then the fluorescence value of mtRP was analyzed by Fluoview 1000 confocal microscope (Olympus, Tokyo, Japan). After 200 s of measurement, 100 μM ATP was added to raise the mitochondrial uptake. The value was measured using excitation wavelength of 436 nm. Alternatively, mitochondrial calcium reporter Rhod2-AM (Thermo Fisher Scientific, Oregon, USA) also was used. H460/Tet-On inducible LETM1 cells were grown until they were 50–70% confluent then were treated with doxycycline to induce LETM1. Cells then were stained with 2 μM Rhod2-AM for 15 min following the manufacturer’s instructions. Cells were washed and stained with 100 nM Mito-tracker green (Thermo Fisher Scientific, Oregon, USA) then observed under microscope.

### Analysis of mPTP dynamics

In order to investigate the effect of LETM1 on mitochondrial permeability transition pore (mPTP) dynamics, MitoProbe Transition Pore Assay Kit (Molecular Probes, Oregon, USA) were employed. This assay uses calcein fluorescence in the presence of CoCl_2_ as an indicator of the calcein retention within mitochondria when the mPTP is in the closed state. CoCl_2_ quenches cytoplasmic calcein fluorescence, rendering the mitochondrial-specific signal. H460 cells were infected with Ad-LacZ or Ad-LETM1 for 24 h. Then the cells were resuspended in pre-warmed Hanks Balanced Salt Solution (HBSS) with Ca^2+^ at a final concentration of 1 × 10^6^ cells/mL. The cells then were stained with 10 nM calcein AM and followed by adding 400 μM CoCl_2_. For the positive control, Ad-LacZ-infected cells were treated with 500 nM ionomycin. Samples were incubated for 15 min at 37 °C and protected from light. Cells were pelleted by centrifugation, re-suspended in 500 μL PBS and filtered into FACS Falcon tubes (Becton Dickinson, Oxford, UK) for flow cytometry analysis. Samples were analyzed using 488 nm excitation and emission filters appropriate for fluorescein with BD FACS Diva software. A sample without added reagents was used for the instrument set up.

### Isolation of mitochondria

H460 cells were washed with PBS and re-suspended in mitochondrial fraction buffer (20 mM Hepes, pH 8.0, 10 mM KCl, 1.5 mM MgCl2, 1 mM EDTA, 250 mM sucrose, 1 mM PMSF, 10 g/mL leupeptin, 10 g/mL aprotinin, and 0.2 mM sodium orthovanadate) for 15 min on ice and then homogenized using 1 ml syringe. Unbroken cells and nuclei were pelleted by centrifugation at 800 g for 10 min at 4 °C. The supernatant was continuously centrifuged at 17,000 g for 10 min at 4 °C and the supernatant was transferred to a new tube to be used as a cytosolic fraction. The pellet was washed with 500 μL of mitochondrial fraction buffer, and used as isolated mitochondrial fraction. Thus isolated mitochondria were lysed using cell lysis buffer supplemented with Protease inhibitor (Sigma Aldrich) on ice for 10 min followed by vortexing. Then the lysates were subjected to centrifugation at 12,000 g for 15 min. The supernatant was transferred into new tubes and analyzed by western blotting.

### Generation of H460/Tet-On inducible LETM1 cells

H460 cells were treated with lentivirus-TET3G and selected with G418 (200 μg/ml) to generate H460-TET3G lung cancer cells. Next, H460-TET3G cells were transiently transfected with pLVX-TRE3G-LETM1 and selected with puromycin (1.25 μg/ml) for H460/Tet-On inducible LETM1 cells. The induction of LETM1 expression was confirmed in the media with 100 ng/ml doxycycline (Doxy) for 24 h.

### Measurement of mitophagy with mito-Keima

The fluorescence protein keima is well known for the distinct fluorescence characteristics of different wavelength based on the surrounding pH environments [25]. Therefore, mitochondria-targeted keima (mt-Keima) has been used for detecting mitophagy in the cells. mt-Keima was prepared as previously described [26]. H460/Tet-On inducible LETM1 cells were transfected with pLESIP-mt-Keima and incubated with 100 ng/ml doxycycline for 24 hr to induce LETM1 overexpression before treating with 25 μM Honokiol or 30 μM CCCP for 8 hr. The cells then were collected then analyzed red and green fluorescence by FACS and confocal microscope. Levels of mitophagic events were calculated by the switching from green to red fluorescence (P2 population).

### Biotinylation of complex proteins by LETM1-APEX2

Desthiobiotin-phenol (DBP) labeling by apurinic/apyrimidinic endodeoxyribonuclease-2 (APEX2) was processed as described in the previous study [27]. HEK293/LETM1-APEX2 stable cells [27] were grown and induced with 500 ng/ml doxycycline at 60–80% confluence. After 24 hr, the medium in plate was changed to fresh growth medium containing 250 µM DBP for 30 min. Afterwards, H_2_O_2_ (Sigma Aldrich H1009) was added at a final concentration of 1 mM, and the plates were gently agitated for 1 min at room temperature. The reaction was then quenched by adding DPBS (Dulbecco’s phosphate-buffered saline) containing 10 mM Trolox, 20 mM sodium azide and 20 mM sodium ascorbate. Then the cells were washed three times with cold quenching solution (DPBS containing 5 mM Trolox, 10 mM sodium azide, and 10 mM sodium ascorbate). Cells were detached using of cold quenching solution and centrifuged at 1500 × g for 5 min at 4 °C. Cells were suspended with fresh cold quenching solution and centrifuged again. DBP-labeling proteins were full-down from 1000 μg of cell-free extracts by using streptavidin bead. After washing the bead, GRP78 and GRP75 were detected by using immune-blotting.

### Human lung cancer tissues

Human lung cancer tissues were obtained by Korea University Guro Hospital of Biobank, a member of the National Biobank of Korea. The experiments using human tissues were authorized by Seoul national University Institutional Review Board (SNUIRB-E1201/001-001).

### Statistical analysis

Quantification of western blot analysis was carried out by using the Image J (1.47) program. Data were expressed as means ± SEM of the three independent experiments and analyzed by Student’s unpaired *t*-test (SPSS version 17.0 software, SPSS Inc.). *p* < 0.05 (*) was considered significant, and *p* < 0.01 (**) was highly significant compared with corresponding control values.

## Results

### Change of autophagic flux by LETM1-overexpression or LETM1-Knockdown in H460 cells

**Fig. 1:**
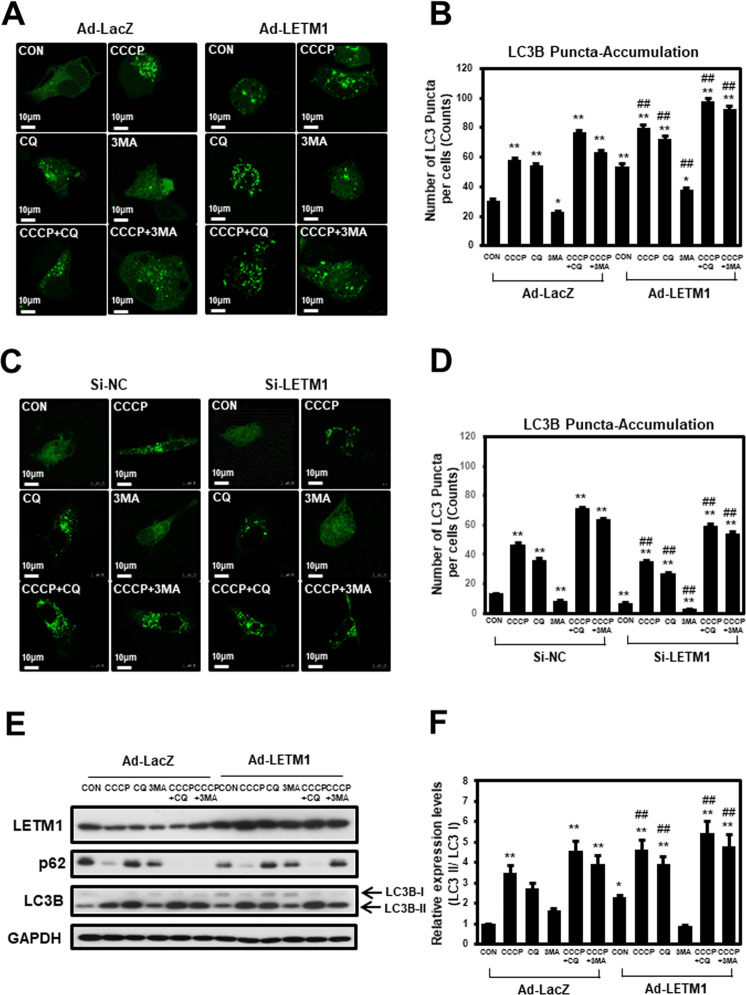
The effects of Leucine zipper-, EF-hand-containing transmembrane protein-1 (LETM1) on autophagy in H460. **A** H460 cells were transfected with GFP-LC3, followed by infection with adenovirus-LacZ (Ad-LacZ) or adenovirus-LETM1 (Ad-LETM1) for 24 h. The cells were then treated with 30 μM carbonyl cyanide m-chlorophenylhydrazone (CCCP), 10 μM chloroquine (CQ), and 10 μM 3-methlyladenine (3MA) for 8 h. The cells were further analyzed with confocal microscopy. The results are representative of three independent experiments. Scale bars represent 10 μm. **B** GFP-LC3 puncta in H460 cells were counted in each condition (*n* = 40). Data are expressed as the mean ± SD using one-way ANOVA with Tukey’s correction for multiple comparisons. *p < 0.05; **p < 0.01, versus the control Ad-LacZ samples, #*p* < 0.05, control Ad-LETM1 versus Ad-LacZ samples. **C** H460 cells were co-transfected with si-RNA negative control of si-RNA LETM1 and GFP-LC3 for 24 h. The si-RNA LETM1 mediated cells were then treated with the indicated compounds. These images are representative of three independent experiments. Scale bars represent 10 μm. **D** GFP-LC3 puncta in H460 cells were counted in each condition (*n* = 40). Data are expressed as the mean ± SD using one-way ANOVA with Tukey’s correction for multiple comparisons. *p < 0.05; **p < 0.01, versus the control si-NC samples, #*p* < 0.05; ##*p* < 0.01, control si-LETM1 versus si-NC samples. **E** H460 cells were infected with Ad-LacZ or Ad-LETM1 for 24 h followed by CCCP, CQ and 3MA treatment for 8 h. The total cell lysates were immunoblotted for LETM1, p62, LC3B, and GAPDH. The results are representative of three independent experiments. **F** Relative densities of each protein were obtained by analyzing the blot using ImageJ software (NIH, Bethesda, MD, USA). The relative expression of LC3B II protein was calculated by normalizing all density values to that of LC3B I in each lane. The results are displayed as mean ± SD and are representative of three independent experiments. *p < 0.05; **p < 0.01.

The effects of LETM1 on autophagic flux in non-small cell lung cancer (NSCLC) cells was evaluated in this study. Control H460 cells infected with LacZ-adenovirus (Ad-LacZ) showed a diffuse cytoplasmic distribution of GFP-LC3B; meanwhile, adenovirus-LETM1 (Ad-LETM1)-infected H460 cells displayed a significant accumulation of GFP-LC3B puncta in confocal microscopic analysis (Fig. 1A, Left of top panel, respectively), indicating enhanced autophagic flux in these cells. This was also seen in cells treated with CCCP (a chemical mitophagy inducer; Fig. 1A, Right of top panel, respectively) and chloroquine (CQ; an inhibitor of the fusion of autophagosomes with lysosomes; Fig. 1A, Left of middle panel, respectively). In addition, LETM1-induced autophagy is sensitive to the classical autophagy inhibitors, wortmannin (WM) and 3-methyladenine (3-MA; Fig. 1A, Right of middle panel, respectively). As expected, the treatment of LETM1-overexpressed H460 cells with CQ markedly increased the accumulation of GFP-LC3B compared to H460 cells with LETM1 expression alone. Statistical analyses of the three independent experiments clearly indicated that LETM1 overexpression was able to promote autophagic flux in H460 cells (Fig. 1B). Similar results were also found in another NSCLC cell line, i.e., A549 cells (Supplementary Fig. 1A, B). Conversely, when LETM1 was knockdown, the opposite results were obtained in H460 cells. LETM1 was knockdown in H460 cells by using siRNA. Control H460 cells mediated with siRNA-control (si-NC) showed a diffuse cytoplasmic distribution of GFP-CL3B; on the other hands, siRNA-LETM1 (si-LETM1)-mediated H460 cells displayed a significant reduction of GFP-LC3B puncta in confocal microscopic analysis (Fig. 1C, Left of top panel, respectively), indicating decreased autophagic flux in these cells. As expected, the mediated si-LETM1 knockdown H460 cells with CCCP or CQ markedly decreased the accumulation of GFP-LC3B compared to H460 cells with si-NC expression cells. GFP-LC3B puncta were decreased when LETM1 was knockdown despite CCCP treatment to induce mitophagy compared to the control (Fig. 1C Right of top Panel, respectively). To further investigate the effects of LETM1 on autophagic influx in H460 cells, changes in the autophagy marker protein LC3B were monitored. As shown in Fig. 1E, F, expression of LC3B-II under the same conditions as Fig. 1A and Supplementary. Fig. 1A, which is recruited to autophagosomal membranes, is increased in LETM1-overexpressed cells. In addition, the expression of p62, a classical receptor of autophagy, is decreased in LETM1-overexpressed cells. This indicates that LETM1 overexpression promotes autophagic flux in H460 cells and LETM1 knockdown attenuates autophagic flux in H460 cells.

### LETM1 overexpression leads to mitophagy in H460 cells

**Fig. 2:**
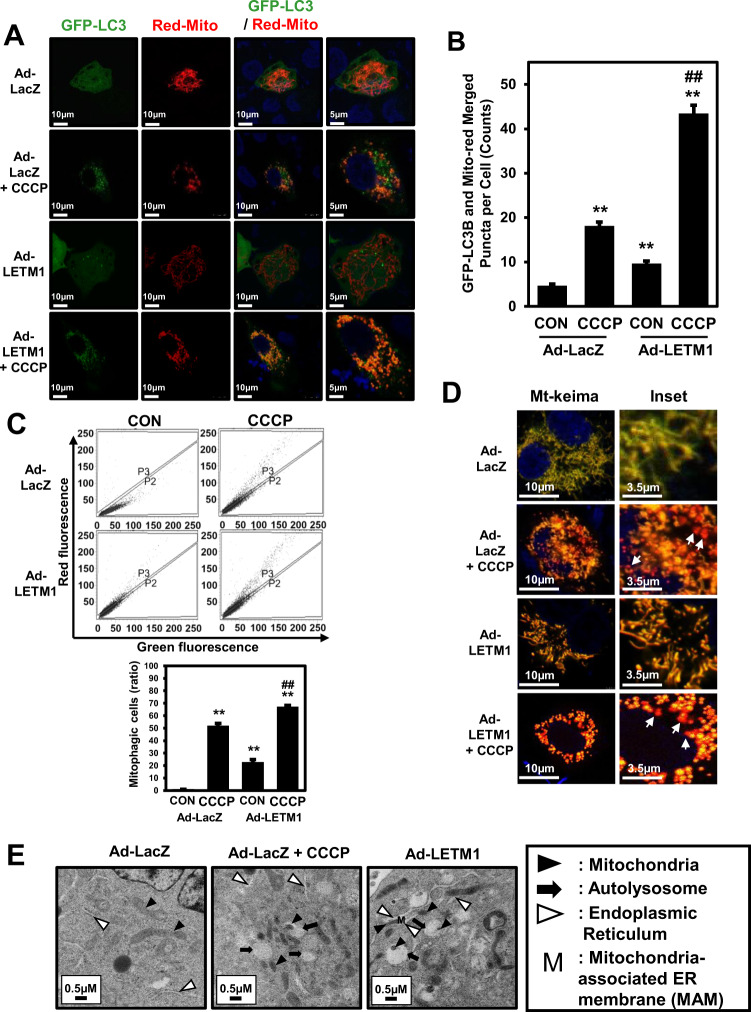
LETM1-mediated mitophagy in H460 cells. **A** H460 cells were transfected with pDs-Red-Mito for 24 h, followed by infection with Ad-GFP-LC3 and Ad-LacZ or Ad-LETM1 for 24 h. For the positive control, cells were treated with 30 μM CCCP for 8 h. These confocal images are representative of three independent experiments. Scale bars represent 10 μm or 5 μm. **B** Merged GFP-LC3 and Red-Mito signals from puncta in H460 cells were counted in each condition (*n* = 40). The results are displayed as mean ± SD and are representative of three independent experiments. *p < 0.05; **p < 0.01, versus the control Ad-LacZ samples, #*p* < 0.05, control Ad-LETM1 versus CCCP Ad-LETM1 sample. **C** H460 cells were transfected with mt-keima for 24 h, followed by infection with Ad-LacZ or Ad-LETM1 for 24 h. Next, these cells are treated CCCP for 8 h. These FACS analysis are representative of three independent experiments. The results are displayed as mean ± SD and are representative of three independent experiments. *p < 0.05; **p < 0.01, versus the control Ad-LacZ samples, #*p* < 0.05, control Ad-LETM1 versus CCCP Ad-LETM1 sample. **D** H460 cells were transfected with mt-keima for 24 h, followed by infection with Ad-LacZ or Ad-LETM1 for 24 h. Next, these cells are treated CCCP for 8 h. These cells are analyzed by confocal microscope. Scale bars represent 10 μm or 3.5 μm. **E** Ad-LacZ or Ad-LETM1-infected H460 cells were analyzed by transmission electron microscopy. Scale bars represent 0.5 μm. Arrowheads: mitochondria; N: nucleus; black arrows: autophagosomes; white arrows: ER.

Based on the previous results (Fig. 1), the potential involvement of LETM1 in mitochondria-specific autophagy was investigated using confocal microscopy and double staining of cells with GFP-LC3B and Red-Mito. H460 cells were transiently transfected with Red-Mito for 24 h, followed by co-infection with Ad-GFP-LC3B and Ad-LacZ or Ad-LETM1 for 24 h (Fig. 2A, first panel and third panel, respectively). To induce mitophagy, Ad-LacZ or Ad-LETM1-infected cells were further treated with CCCP for 8 h (Fig. 2A, second panel and fourth panel, respectively). The mitochondria labeled with Red-Mito were fragmented and appeared as dot-like structures colocalized with GFP-LC3B puncta in LETM1-overexpressed cells, similar to the CCCP-treated cells (Fig. 2A). Indeed, the co-localization analysis (Supplementary Fig. 2) and merged puncta quantification (Fig. 2B) indicated that mitophagic process is occurred in these cells.

To reliably determine whether mitophagy is induced in H460 cells by LETM1, these cells were tested using the mt-Keima expression vector [26], and in-vivo mitophagy indicator. Using the flow cytometry analysis method, LETM1-overexpressed cells exhibited marked change from green to red color. It has been shown the similar pattern as Ad-LacZ and CCCP treat cells (Fig. 2C). In addition, as a results of confirming using confocal under the same conditions, red puncta were confirmed in LETM1-overexpressed cells similar to Ad-LacZ and treated CCCP cells (Fig. 2D). These data indicated that LETM1 can promote Mitophagic clearance in H460 cells.

The TEM images showed that the control cells exhibited mitochondria (black arrowheads) with a normal morphology (long tubular phenotypes), whereas the cells with LETM1 overexpression showed fragmented mitochondria appearing as small dot-like structures, the majority of which were in close proximity to autophagosomes (black arrows); they were ultimately engulfed by an increasing accumulation of autophagosomes, as also observed in the cells treated with CCCP (Fig. 2E). The formation of autophagosomes and engulfment of fragmented mitochondria occurred in close proximity to the endoplasmic reticulum (ER; white arrowheads; Fig. 2E). These results further confirmed that overexpression of LETM1 led to mitochondrial dysmorphology and formation of the MAM, followed by the subsequent clearance of these mitochondria through mitophagy in H460 cells.

### Mitochondrial function is defective in LETM1-overexpressed H460 cells

**Fig. 3:**
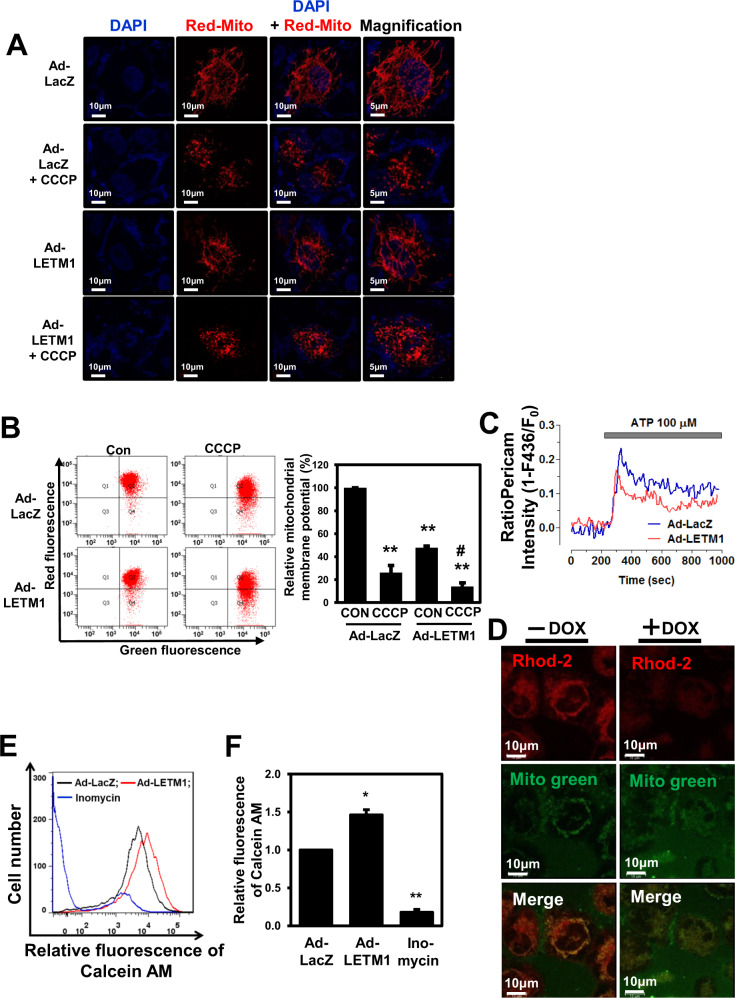
Mitochondrial dysfunction in LETM-overexpressed H460 cells. **A** H460 cells were transfected with pDs-Red-Mito, followed by infection with Ad-LacZ or Ad-LETM1 for 24 h. Ad-LacZ-infected cells were treated with 30 μM CCCP for 8 h. These results are representative of three independent experiments. Magnified images (× 100) are indicated (left panel). Scale bars represent 10 μm or 5 μm. **B** H460 cells were infected with Ad-LacZ or Ad-LETM1 for 24 h. Ad-LacZ-infected cells were treated with 30 μM CCCP for 15 min and cells were then incubated with 10 ng/mL JC-1 for 30 min. Mitochondrial membrane potential (MMP) was assayed with FACS; fluorescent density is shown. The images are representative of three independent experiments. The relative values of the RFP signal (PE-A) and GFP signal (FITC) were calculated and are plotted as mean ± SD of three independent experiments. **p < 0.01. **C** H460 cells were transfected with mitochondria-targeted ratio-pericam (mtRP) for 24 h, followed by infection with Ad-LacZ or Ad-LETM1 for 24 h. Calcium uptake was initiated by adding 100 μM ATP after 200 s, indicated by the gray bar. Fluorescence values for mtRP were analyzed by a FluoView confocal microscope (Olympus, Tokyo, Japan). The images are results are representative of three independent experiments. **D** H460/Tet-On-inducible LETM1 cells were treated with doxycycline to induce LETM1 expression for 24 h. Cells then were stained with Rhod-2 AM, as a calcium, indicator and MitoTracker Green. The confocal images are representative of three independent experiments. Scale bars represent 10 μm. **E** H460 cells were infected with Ad-LacZ or Ad-LETM1 for 24 h. The infected H460 cells were stained with calcein AM followed by FACS analysis. Ionomycin was used as an activator of mitochondrial permeability transition pores (mPTPs). The images are representative of three independent experiments. **F** The relative values of calcein AM are plotted as mean ± SD of three independent experiments. *p < 0.05; **p < 0.01.

First we evaluated the effects of LETM1 on mitochondrial morphology in H460 cells. As expected, overexpression of LETM1 led to disruption of the mitochondrial tubular network and mitochondrial fragmentation, resulting in the accumulation of small dot-like phenotypes as also observed in the cells treated with CCCP (Fig. 3A).

Additionally, Twig and colleagues demonstrated that depolarized daughter mitochondria produced after mitochondrial fission events are unable to continue with the normal cycle of fusion and are degraded by mitophagy [16]. Therefore, we next monitored mitochondrial membrane potential (MMP) using JC-1 dye in LETM1-overexpressed cells, since LETM1 overexpression led to mitochondrial fragmentation and promoted mitophagy in H460 cells (Figs. 1 and 2). The LETM1-overexpressed cells had markedly reduced MMP compared to the control cells, similar to the CCCP-treated cells (Fig. 3B).

LETM1 has been reported to be a mitochondrial Ca^2+^/H^+^ antiporter, which can extrude Ca^2+^ from the matrix under certain physiological conditions to maintain equilibrium between the matrix and intermembrane space [20, 28]. In this regards, the changes in mitochondrial Ca^2+^ levels in H460 cells were measured using a mitochondria-targeted ratio-pericam plasmid. As shown in Fig. 3C, ATP-induced mitochondrial Ca^2+^ uptake was reduced in LETM1-overexpressed cells compared to control cells. To further investigate the effects of LETM1 on the control of mitochondrial Ca^2+^, H460/Tet-On-inducible LETM1 cells were prepared. LETM1 overexpression induced by doxycycline was confirmed by immunoblotting (Fig. 5C). Mitochondrial Ca^2+^ levels, detected by red staining with Rhod2-AM (another mitochondrial calcium reporter), were decreased in doxycycline-treated H460/Tet-On-inducible LETM1 cells (Fig. 3D), suggesting that LETM1 overexpression reduced Ca^2+^ levels in mitochondria.

Under stressful cellular conditions, mitochondrial Ca^2+^ overload is a key factor in mPTP opening, inducing large-scale mitochondrial alterations that ultimately lead to cell death or apoptosis [29]. To investigate possible changes in mPTP dynamics caused by LETM1 overexpression, H460 cells were infected with Ad-LacZ or Ad-LETM1 for 24 h. Then, mPTP dynamics were analyzed using a Mito Probe Transition Pore assay kit and FACS; inomycin, an activator of mPTP, was used as a positive control. Consistent with our previous results regarding mitochondrial Ca^2+^ reduction (Fig. 3C, D), LETM1-overexpressed cells exhibited higher retention of calcein AM fluorescence after treatment with CoCl_2_ compared to control Ad-LacZ-infected cells (Fig. 3E, F). These results indicate a closed mPTP configuration in cells overexpressing LETM1. Interestingly, the respiration capacity seemed to be unaffected in both cell types, since the cell oxygen consumption rate (OCR; Supplementary Fig. 3A) and configuration of oxidative phosphorylation (OXPHOS) complexes (Supplementary Fig. 3B) were not changed in either the Ad-LacZ- or Ad-LETM1-infected H460 cells (Supplementary Fig. 3).

### GRP78 appears to be an interacting partner of LETM1 and contributes to mitophagy

**Fig. 4:**
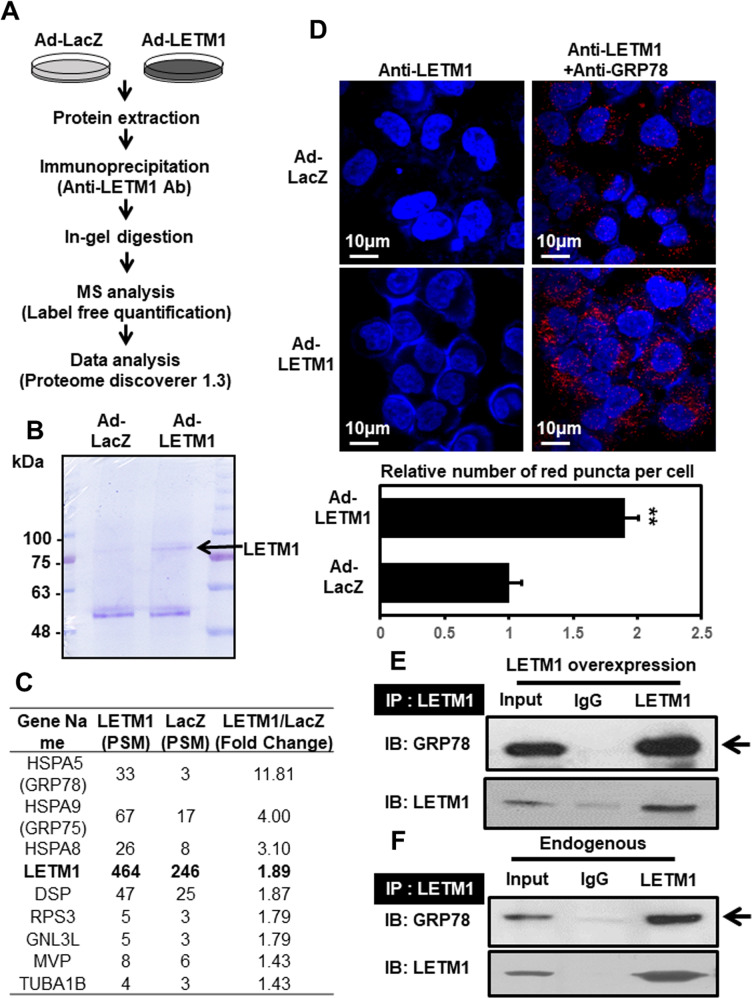
Putative interacting partners of LETM1. **A** The schematic procedure for the experiments. H460 cells were infected with Ad-LacZ or Ad-LETM1 for 24 h. Total cell lysates were immunoprecipitated with anti-LETM1 antibodies and separated by sulfate-polyacrylamide gel electrophoresis (SDS-PAGE). Each lane of samples was cut equally and analyzed by mass spectrometry. **B** Immunoprecipitated LETM1 was resolved by 12% preparative SDS-PAGE and then stained with Coomassie blue. LETM1 is indicated by black arrows. **C** The candidate proteins that bound to LETM1 and were detected by mass spectrometry are listed. **D** H460 cells were seeded on coverslips and then infected with Ad-LacZ or Ad-LETM1 for 24 h. The cells were incubated with anti-LETM antibody, or a combination of anti-LETM1 and anti-glucose-regulated protein 78 kDa (GRP78) antibodies, followed by a Duolink PLA. The interaction is represented by the red signal under confocal microscopy (upper panel) and quantification of red puncta per cell (lower panel). **E** HeLa cells were transfected with pCMV5-Myc-LETM1 for 48 h. The immunoprecipitation products with anti-LETM1 antibodies were immunoblotted with anti-GRP78 antibodies (top panel). LETM1 overexpression was confirmed by immune blot analysis with anti-LETM1 antibodies (bottom panel). **F** Endogenous interactions of GRP78 and LETM1 were also monitored in HeLa cells. The results are representative of three independent experiments.

To identify potential interacting partners of LETM1 that might play roles in LETM1-mediated mitophagic clearance, LETM1-overexpressed H460 cells were used for preparing immunoprecipitants with anti-LETM1 antibodies. An assay for LETM1 binding partners was then employed (Fig. 4A). LETM1 was pulled down well (Fig. 4B) and able to bind with 58 candidates (Fig. 4C). Among them, GRP78 (HSPA5) and GRP75 were found to be the most promising binding partners, showing the highest fold changes, of 11- and 4-fold, respectively (Fig. 4C). These results were further confirmed by the protein-protein interactions observed using a Duolink-proximity ligation assay (PLA) (Fig. 4D–F). Taken together, these results indicate that GRP78 is an interacting partner of LETM1 in H460 cells.

### GRP78 activity is required for LETM1-mediated mitochondrial dysfunction and complex formation in the MAM

**Fig. 5:**
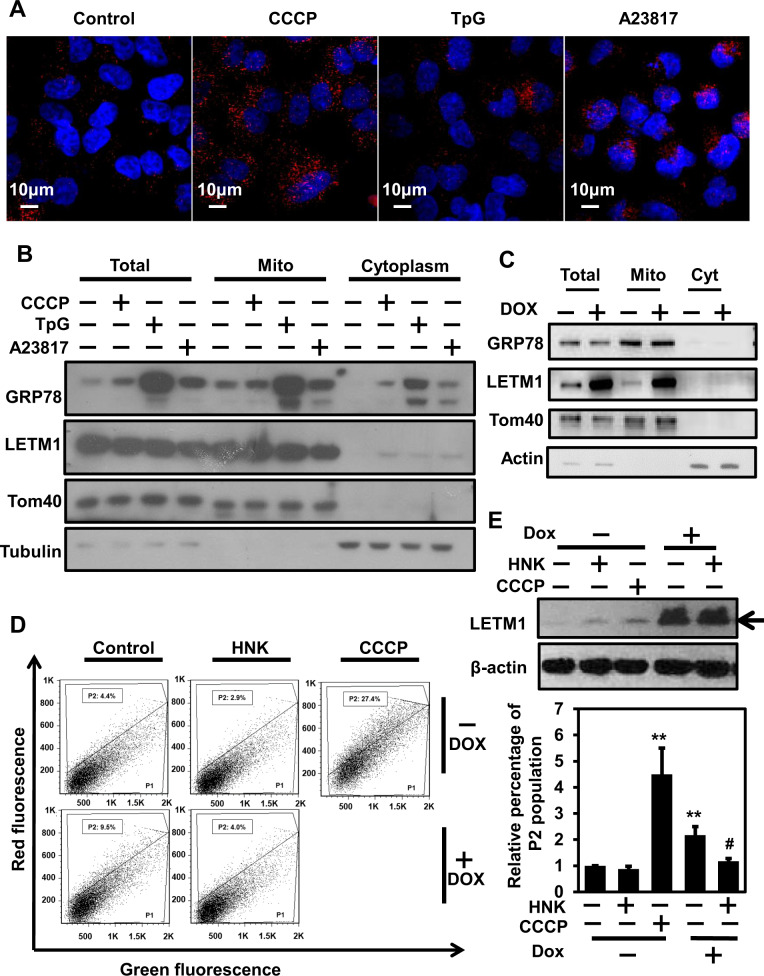
Regulation of the LETM1/GRP78 complex via various stimulators. **A** H460 cells were plated on coverslips and treated with 30 μM CCCP, 1 μM thapsigargin (TpG), or 5 μM A23817 for 8 h. The interaction between LETM1 and GRP78 was monitored by a Duolink PLA. The interaction is represented by the red signal under confocal microscopy. **B** H460 cells were treated with the indicated compounds for 8 h. Total cell lysates and isolated mitochondria fractions were analyzed by western blotting with anti-LETM1, anti-GRP78, anti-Tom40, and anti-tubulin antibodies. These results are representative of three independent experiments. **C** H460/Tet-On-inducible LETM1 cells were seeded onto 10-cm plates. LETM1 expression was induced by incubating the cells with 100 ng/ml of doxycycline for 24 h. The cells were fractionated and analyzed by western blotting with the indicated antibodies. These results are representative of three independent experiments. **D** H460/Tet-On-inducible LETM1 cells were transfected with mt-Keima expression vector and incubated with 100 ng/ml doxycycline for 24 h to induce LETM1 overexpression, before being treated with 25 μM honokiol (HNK) or 30 μM CCCP for 8 h. The cells were then collected by trypsinization and analyzed for red and green fluorescence by FACS. Sub-population P1 contained a high green and low red signal; sub-population P2 contained a high red and low green signal, indicating mitophagic cells. These results are representative of three independent experiments. **E** The relative percentage of the P2 population was plotted as mean ± SD of three independent experiments. *p < 0.05; **p < 0.01. LETM1 overexpression was confirmed by the immunoblot analysis with anti-LETM1 antibodies (top panel). These results are representative of three independent experiments.

Based on the observation that LETM1 can bind to GRP78 (Fig. 4D–F), we next evaluated the regulation of the LETM1/GRP78 complex by treating cells with various stimulators (CCCP for mitophagy; thapsigargin (TpG) for ER stress; A23817 for Ca^2+^ ionophore-induced activation). Each stimulator was associated with distinct localization of the LETM1/GRP78 complex in H460 cells. Similar to the localization of the LETM1/GRP78 complex in LETM1-overexpressed H460 cells (Fig. 4D), CCCP stimulation resulted in localization of the LETM1/GRP78 complex distributing throughout the cellular cytoplasm (Fig. 5A, second panel), while treatment of cells with TpG or A22817 resulted in localization of the LETM1/GRP78 complex close to the nucleus (Fig. 5A, final two panels). Interestingly, mitochondria-localized GRP78 was increased by TpG and A23817 treatment, but not by CCCP treatment (Fig. 5B).

To further investigate the effects of LETM1 on GRP78 translocation to the mitochondria, H460/Tet-On-inducible LETM1 cells were employed. Similar to the results for CCCP-treated samples (Fig. 5B), there was no significant change in mitochondrial GRP78 levels in LETM1-overexpressed H460 cells, suggesting that while the LETM1/GRP78 complex does not require mitochondrial translocation of GRP78, it may be a core component of the MAM, which leads to mitochondrial dysfunction (Fig. 2E). To further evaluate the roles of GRP78 in LETM1-induced mitophagy in H460 cells, the GRP78 inhibitor, Honokiol (HNK; [30]), and mt-Keima expression vector were used. CCCP-mediated mitophagy was obvious based on the increase in P2 (mitophagic population) size (by 27.4%; Fig. 5D, top row, third panel) in mt-Keima-expressed H460 cells compared to control cells (4.4%). As expected, doxycycline-induced LETM1 expression (Fig. 5E, top panel) led an increase in P2 population size (9.5%), whereas pretreatment with HNK blocked LETM1-induced mitophagy (4.0%) such that the level thereof was similar to that in control cells (4.4%). Statistical analysis of P2 populations (Fig. 5E, bottom panel) clearly suggest that GRP78 activity is required for LETM1-mediated mitophagy in H460 cells.

### GRP75 is important for LETM1/GRP78-mediated mitophagy and MAM

**Fig. 6:**
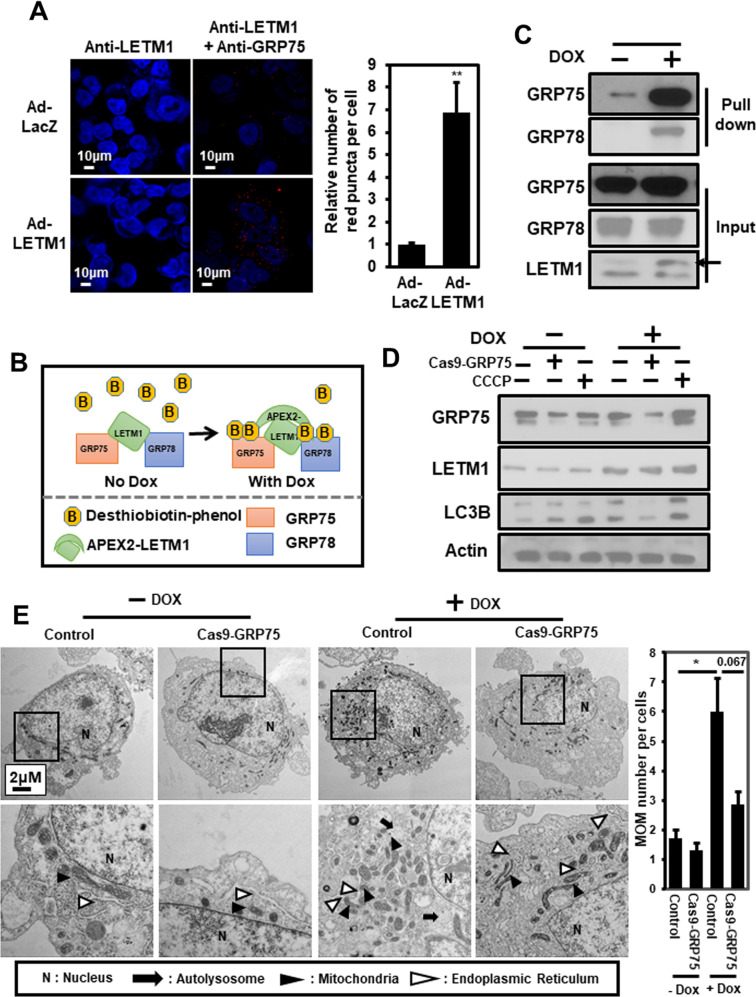
Effects of glucose-regulated protein 75 kDa (GRP75) on LETM1-mediated autophagy. **A** H460 cells were seeded on coverslips and then infected with Ad-LacZ or Ad-LETM1 for 24 h. The cells were incubated with anti-LETM antibody alone, or anti-LETM1 and anti-GRP75 antibodies, followed by a Duolink PLA. The interaction is represented by the red signal under confocal microscopy (left panel) and quantification of red puncta per cell (right panel). **B** Schematic showing the procedure for the desthiobiotin-phenol (DBP) labeling experiments. **C** LETM1-APEX2 in stable HEK293 cells were treated with doxycycline for 24 h, followed by the DBP labeling experiment as described in the Material and Methods. DBP-GRP75 and DBP-GRP78 were pulled down by streptavidin beads and detected by western blotting. The arrow points APEX2-LETM1 band. **D** H460/Tet-On-inducible LETM1 cells were seeded onto 10-cm plates overnight. CRISPR/Cas9-GRP75 plasmid was used to knock down GRP75. LETM1 expression was induced by incubating the cells with 100 ng/ml doxycycline for 24 h. For the positive control, cells were treated with 30 μM CCCP for 8 h. The cells were lysed and analyzed by western blotting with the indicated antibodies. These results are representative of three independent experiments. **E** CRISPR/Cas9-GRP75 plasmid was used to knock down in H460/Tet-On-inducible LETM1 cells. For the positive control, cells were treated with 30 μM CCCP for 8 h. These cells were analyzed by transmission electron microscopy. Scale bars represent 2 μM. N: nucleus; block arrows; autophagosomes; arrowheads: mitochondria; white arros; ER; The quantification graph was calculated from 7 different cells each condition. Mitochondrial-ER association point was counted per single cell. *p < 0.05.

**Fig. 7:**
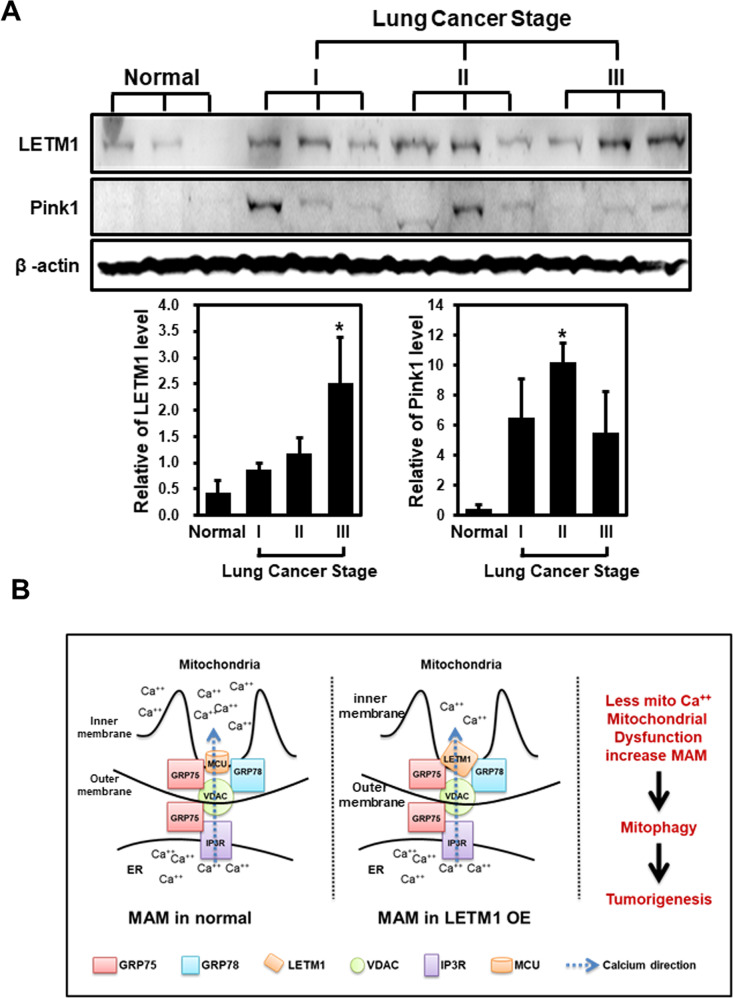
A suggested model of LETM1 function in tumorigenesis. **A** The human samples were collected and lysate and following by a western blot analysis with anti-LETM1, anti-PINK1 and anti-Actin antibodies. The blots were then quantified by using ImageJ software (NIH, Bethesda, MD, USA). The relative expression of indicated protein (LETM1, PINK1) was calculated by normalizing all density values to that of actin in each lane. The results are displayed as mean ± SD and are representative of three independent samples. *p < 0.05. The proposed model of the role of LETM1 overexpression in mitophagy and tumorigenesis. **B** Overexpression of LETM1 induces mitochondria-associated ER membrane (MAM) by interacting with GRP78 and/or GRP75 and consequently reducing Ca^++^ uptake. The formation of MAM, as triggered by LETM1 overexpression, leads to mitochondrial dysfunctions such as fragmentation, defects in Ca^++^ and mPTP homeostasis, and loss of MMP, resulting in mitophagy. Mitophagy induced by LETM1 overexpression in lung cancer cells is advantageous for tumorigenesis.

Since there was no significant alteration in the mitochondrial translocation of GRP78 following CCCP treatment (Fig. 5B), or of LETM1 overexpression (Fig. 5C), the involvement of GRP75 (another putative binding partner for LETM1; see Fig. 4C) in LETM1-mediated mitophagy was investigated. Duolink-PLA confirmed the basal interaction between LETM1 and GRP75 in Ad-LacZ-infected H460 cells, indicated by red-dots signal of PLA (Fig. 6A, top panel). Similar to GRP78, a strong interaction was also found in Ad-LETM1-infected cells (Fig. 6A, bottom panel, 8-fold increase). To further investigate the complex formation of LETM1, GRP75, and GRP78, a biotin labeling assay was employed, as described previously [27]. Peroxidase conjugation of LETM1 to APEX2 (APEX2-LETM1) was induced by doxycycline in HEK293/LETM1-APEX2 stable cells, as described previously by Lee et.al. In the presence of desthiobiotin-phenol (DBP), APEX2-LETM1 induced biotinylation of LETM1-neighboring proteins, which could then be pulled down by streptavidin beads (Fig. 6B). As expected, both GRP75 and GRP78 were conjugated with DBP and pulled down with the streptavidin beads following doxycycline induction of APEX2-LETM1 in stable HEK293 cells (Fig. 6C). This observation indicates that the LETM1/GRP78/GRP75 complex could form in cells. To further evaluate the effects of GRP75 on LETM1-mediated autophagy, GRP75 was depleted by using the CRISPR/Cas9 system in H460 cells. Interestingly, GRP75-depletion blocked LETM1-induced autophagy, indicated by the reduction of LC3B/II in CRISPR/Cas9-GRP75-transfected H460 cells (Fig. 6D). To further confirm the effect of GRP75, the ultrastructure of cells was analyzed using TEM. As shown in Fig. 2E, the cells with doxy-induced LETM1 overexpression showed more mitochondria fragmentation compared to the control cell. Interestingly, GRP75-depletion in LETM1 overexpression cells were decreased mitochondria autophagy and increased tubular morphology (black arrowheads). It was also reduced the formation of MAM between mitochondria and ER (white arrowheads). These results suggest that GRP75 is necessary for LETM1-induced autophagy. Indeed, both levels of LETM1 and PINK1 were elevated in the human lung carcinoma implicating for the association of LETM1-mitophagy and lung cancer progression and development (Fig. 7A). In summary, LETM1 might be a key factor in the early stages of lung tumor development, by triggering mitophagy and aiding formation of the MAM via the GRP78/GRP75 interaction (Fig. 7B).

## Discussion

LETM1 serves as an integral mitochondrial protein located on the matrix side of the inner membrane [31]; its function in maintaining mitochondrial morphological homeostasis and dynamics has been well established [32]. Studies using a knock-down model of LETM1 have demonstrated that LETM1 depletion leads to fragmented dot-like mitochondria with swollen cristae and loss of the tubular mitochondrial network [21, 31]. However, consistent with the observations by Piao and colleagues [18] our experiments demonstrated that LETM1 overexpression results in fragmented small dot- or doughnut-like mitochondria in NSCLC cells (Fig. 3A).

Earlier studies using LETM1 overexpression models demonstrated its role in mitochondrial dysfunction, dysmorphology, apoptosis, and cancerous metabolism [18, 19, 33, 34]. Similarlly, LETM1-mediated mitochondrial dysmorphology and dysfunction leads to an enhancement of general autophagy in NSCLC cells (Fig.1). Moreover, LETM1 overexpression resulted in mitochondrial clearance by mitophagy (Fig. 2A, B, Supplementary Fig. 2, Fig. 5D, E and Fig. 2E). Most strikingly, in the TEM analysis indicated that the overexpression of LETM1 led to mitochondrial dysmorphology and formation of the MAM, followed by subsequent clearance of these mitochondria through mitophagy in H460 cells (Fig. 2E).

It was suggested that LETM1 itself functions as a Ca^2+^/H^+^ antiporter that exchanges Ca^2+^ for H^+^ at submicromolar cytosolic concentrations to maintain mitochondrial Ca^2+^ homeostasis, dependent upon the pH gradient [28]. In the reconstitution study with purified LETM1 and liposomes, LETM1-mediated Ca^2+^ exchange was Ca^2+^-dependent and Ca^2+^ was extruded from the matrix under physiological conditions to maintain the equilibrium condition between the matrix and intermembrane [20]. In this regards, a reduction in mitochondrial calcium levels was found in LETM1-overexpressed H460 cells compared to control cells (Fig. 3C, D). Mitochondrial Ca^2+^ overload can result in the opening of mPTPs, subsequently leading to depolarization and transient flushing of Ca^2+^ ions from the mitochondria [29, 35]. Previously, a closed mPTP configuration was found in lymphoblastoid cells derived from WHS patients who exhibited LETM1 depletion [36]. However, LETM1 overexpression resulted in the closed mPTP configuration, unlike what was seen in the controls, in our study (Fig. 3E, F). A possible explanation for this discrepancy is that LETM1 could interact with other, as-yet unknown mediators to regulate mPTP dynamics in the mitochondrial inner membrane of H460 cells.

Attempts to find the key molecular player in LETM1-mediated mitophagy via conventional immunoprecipitation and mass-spectrometric analysis (Fig. 4A–C) have revealed various putative interacting proteins, including GRP78 and GRP75 which later on was confirmed to interact with LETM2 endo-and exo-genously (Fig. 4D–F). Both GRP78 and GRP75 belong to Heat shock protein 70 (HSP70) chaperones and are well known as the master regulators facilitating the protein degradation process involving in immune function regulation, cell cycle, survival and death [37, 38]. Interestingly, GRP78 was recently proven to be an autophagic marker [39, 40]. Moreover, the endogenous complex formation of LETM1 and GRP78 was observed to be enhanced under mitophagic conditions (Fig. 5A) without additional mitochondrial localization of GRP78 (Fig. 5B, C). In addition, GRP78/GRP75 facilitates the ER-mitochondrial interaction [37], which was clearly observed in TEM images of LETM1-overexpressed cells (Figs. 2E, 6E). Therefore, ER-GRP78 and mitochondrial LETM1 can bind to each other to create a MAM between the ER and mitochondria. MAM is a subdomain of the ER that communicates with the mitochondria, both physically and biochemically [41–44].

Similar to GRP78, GRP75 can also interact with LETM1 (Figs. 4 and 6). Interestingly, both GRP78 and GRP75 interact with VDAC, an outer mitochondrial membrane protein involved in the MAM and Ca^2+^ homeostasis [45, 46]. It is commonly accepted that the main structure responsible for ER-mitochondrial calcium transfer at the MAM is composed of an IP3R on the ER, VDAC on the mitochondrial outer membrane, and mitochondrial Ca^2+^ uniporter (MCU) on the mitochondrial inner membrane (Fig. 7B, left panel). Upon activation of IP3R, Ca^2+^ at the ER is taken up into mitochondria, first by VDAC and then by MCU [46–48]. This transduction was weakened by the presence of LETM1 in the MAM (Figs. 3C, D and 7B). Based on these observations, we proposed a LETM1-constituted MAM model, in which LETM1 serves as a “MAM anchor” in mitochondria by interacting with GRP78 and/or GRP75, thereby abolishing MCU-modulated Ca^2+^ flux to the mitochondria. Indeed, MCU drives rapid and massive calcium entry, but only under high cytosolic Ca^2+^ concentrations (> 10 µM) at the contact sites of the mitochondria and ER; meanwhile, LETM1 is responsible for the slow entry of calcium into the mitochondria at low cytosolic Ca^2+^ concentrations (> 100 nM) [49]. Moreover, LETM1 is bidirectional and can extrude Ca^2+^ [20]; therefore, the Ca^2+^ uptake activity mediated by LETM1 is limited to the site of contact with the ER, leading to a reduction in mitochondrial calcium levels (Fig. 3C, D). Remarkably, CCCP treatment resulted in similar phenomena as LETM1 overexpression, including LETM1-GRP78 interaction, mitochondrial GRP78 localization, MMP, and mitophagy induction (Figs. 1–3 and 5), leading to enhancement of ER-mitochondrial contact [22] and a reduction of mitochondrial Ca^2+^ [50]. Furthermore, the increased ER-mitochondria contact facilitated autophagy and mitophagy [48, 51]. Thus, MAM constituted by LETM1-GRP78 may be a key factor in promoting ER-mitochondria signal transduction, and thus formation of MAM, consequently resulting in mitochondrial dysfunction characterized by an abnormal Ca^2+^ balance, closed mPTP configuration, enhanced mitochondrial fragmentation, loss of MMP (Fig. 3) and, eventually, LETM1-mediated mitophagy (Fig. 2 and Supplementary Fig. 2).

Interestingly, GRP78 has been shown to be highly induced in poorly perfused solid tumors due to microenvironmental factors including hypoxia, acidosis, and glucose deprivation. High levels of GRP78 contribute to the hallmarks of phenotypic cancer, including apoptosis resistance, immune escape, metastasis, and angiogenesis [52]. Likewise, mitophagy is advantageous to cancer cells for removal of the damaged mitochondria generated during tumorigenesis and disease progression [53, 54]. Therefore, LETM1 could bind to GRP78 and/or GRP75, leading to MAM formation and facilitation of mitophagy (Fig. 6E). This implies that LETM1 may have a role in tumorigenesis and/or disease progression, as illustrated in Fig. 7. Thus, future studies should concentrate on the relationship between cancer progression and LETM1, to gain new insight into novel strategies for cancer therapy and/or diagnosis.

## Supplementary information

check list

SUPPLEMENTAL MATERIAL, SUPPLE FIGURE LEGENDS

Supple figure 1

Supple figure 2

Supple figure 3

Dataset Western blot

Dataset 1B

Dataset 1D

Dataset 1F

Dataset 2B

Dataset 2C

Dataset 3B

Dataset 3F

Dataset 4D

Dataset 5D

Dataset 6A

Dataset 6E

Dataset 7A

## Data Availability

All data generated or analyzed during this study are included in this published article (and its supplementary information files).
