# Supplementary material for: Emerging role of LETM1/GRP78 axis in lung cancer
Source: Cell Death Dis. 2022 Jun 10;13(6):543. doi: 10.1038/s41419-022-04993-5 (PMC9184611; doi:10.1038/s41419-022-04993-5)
Supplement: Supplementary file 2 — SUPPLEMENTAL MATERIAL, SUPPLE FIGURE LEGENDS [file 41419_2022_4993_MOESM2_ESM.docx]

**SUPPLEMENTARY DATA**

**Material and Methods**

**Cell culture and stimulation.** A549 cells were maintained in medium (DMEM) supplemented with 10% FBS, 1% Antibiotics-Antimycotics. These cells were transiently transfected using Lipofectamine (Invitrogen, California, USA) or jet-PEI (Polyplus, New York, USA) reagents following the instructions provided by manufacturers. Adenovirus (Ad)-LacZ and Ad-LETM1 were infected for 24 hr. These cells were treated with 30 μM CCCP for 8 hr, 10 μM CQ for 6 hr, 1 μM WM for 2 hr, 25 μM HNK for 8hr or 10 μM 3-MA for 2 hr.

**Mitochondrial morphology analysis with confocal microscopy** A549 cells were grown on glass coverslips until they were 50–70% confluent, followed by transfection of cells with pDsRed-Mito constructs of GFP-LC3 constructs by using Lipofectamine or jet PEI reagents, followed by infection with the combination of proper adenovirus (Ad-LETM1 or Ad-LacZ). After 24 hr, the cells were fixed in 4% paraformaldehyde at room temperature for 10 min and permeabilized in 0.2% Triton X100 for 15 min at room temperature. Then the coverslips were mounted with Vectashield (Vector Laboratories, Burlingame, CA) and visualized using a Zeiss confocal microscope.

**Blue Native-Polyacrylamide Gel Electrophoresis (BN-PAGE).** BN-PAGE was performed using the NativePAGETM Novex® Bis-Tris Gel system (Invitrogen) according to the manufacturer’s instructions. Briefly, 50 ug of isolated mitochondria was solubilized using NativePAGE sample buffer supplemented with 0.5% n-dodecyl-β-D-maltoside. After 30 min incubation on ice, the suspensions were centrifuged at 20,000 x g for 10 min at 4°C. The resulting supernatants were loaded onto a NativePAGE Novex 3–12% Bis-Tris gel. After running, the gel was transferred to a PVDF membrane using the iBlot TM Gel Transfer System (Invitrogen). The membrane was fixed with 8% acetic acid and washed with distilled water. After overnight drying, the membrane was de-stained with methanol and incubated in blocking solution for 30 min at room temperature. For BN-PAGE, the Anti- OXPHOS Complex Kit (Invitrogen) primary antibody cocktail was used. After incubation in the primary antibody dilution, the membrane was washed and detected using the WesternBreeze® (Invitrogen) Chromogenic Western Blot Immunodetection Kit according to the manufacturer’s instructions.

**Oxygen Consumption Rate** The mitochondrial oxygen consumption rate (OCR) was measured using a Seahorse XF-24 extracellular flux analyzer (Seahorse Bioscience). On the day before the experiment, the sensor cartridge was placed into the calibration buffer supplied by Seahorse Bioscience and incubated at 37°C in a non-CO2 incubator. MEFs were cultured on Seahorse XF-24 plates at a density of 20,000 cells per well. Cells were washed and incubated with assay medium (DMEM without bicarbonate) at 37°C in a non-CO2 incubator for 1 hr. All media and injection reagents were adjusted to pH 7.4 on the day of the assay. Three baseline measurements of OCR were taken before sequential injection of mitochondrial inhibitors. Three readings were taken after each addition of mitochondrial inhibitor before injection of the subsequent inhibitors. The mitochondrial inhibitors used were oligomycin (2 μg/ml), CCCP (5 μM), and rotenone (1 μM). OCR was automatically calculated and recorded by the Seahorse XF-24 software. After the assays, the plates were saved, and protein levels were measured for each well to confirm equal cell numbers per well. The percentage of change compared with basal rates was calculated as the value of the change divided by the average value of baseline readings.

**Statistical Analysis.** Quantification of western blot analysis was carried out by using the Image J (1.47) program. Data were expressed as means ± SEM of the three independent experiments and analyzed by Student's unpaired t-test (SPSS version 17.0 software, SPSS Inc.). p<0.05 (*) was considered significant, and p<0.01 (**) was highly significant compared with corresponding control values.

**Figure Legends**

**Supplementary Figure S1. The autophagic flux by LETM1-overexpression in A549 cells** (A) A459 cells were transfected with GFP-LC3 followed by infection with Ad-LacZ or Ad-LETM1 for 24 h. The LETM1-infected cells were then treated with the indicated compounds. These images are representative of three independent experiments. Scale bars represent 10 μm. (B) GFP-LC3 puncta in A549 cells were counted in each condition (n = 40). Data are expressed as the mean ± SD using one-way ANOVA with Tukey’s correction for multiple comparisons. * p < 0.05; ** p < 0.01, versus the control Ad-LacZ samples, # p < 0.05, control Ad-LETM1 versus Ad-LacZ samples * p < 0.05; ** p < 0.01. # p < 0.05, control Ad-LETM1 versus Ad-LacZ samples

**Supplementary Figure S2. Co-localization analysis from figure 2 by using Image J software.** A bar on each image was draw and then graphed the intensity of the color appearing on the bar. When green and red overlap each other, graphs of two colors show similar patterns, otherwise different patterns. The co-localized area was marked with a black box. This method will once again demonstrate that merged puncta are highly induced when LETM1 is overexpressed

**Supplementary Figure S3. Changes of mitochondrial respiration and OXPHOS complexes in Ad-LacZ/Ad-LETM1 infected H460 cells** (A) H460 cells were infected with Ad-LacZ or Ad-LETM1 for 24 hrs before measuring the oxygen consumption rate by using Seahorse XF-24 extracellular flux analyzer. Oligomycin, CCCP and Rotenone consequently add to the cells to investigate OCR in different conditions. (B) Mitochondrial fraction was isolated from Ad-LacZ and Ad-LETM1 infected H460 cells to assess the steady-state levels of 5-OXPHOS complexes by Blue native PAGE.
