# Supplementary figures and images for: Emerging role of LETM1/GRP78 axis in lung cancer
Source: Cell Death Dis. 2022 Jun 10;13(6):543. doi: 10.1038/s41419-022-04993-5 (PMC9184611; doi:10.1038/s41419-022-04993-5)

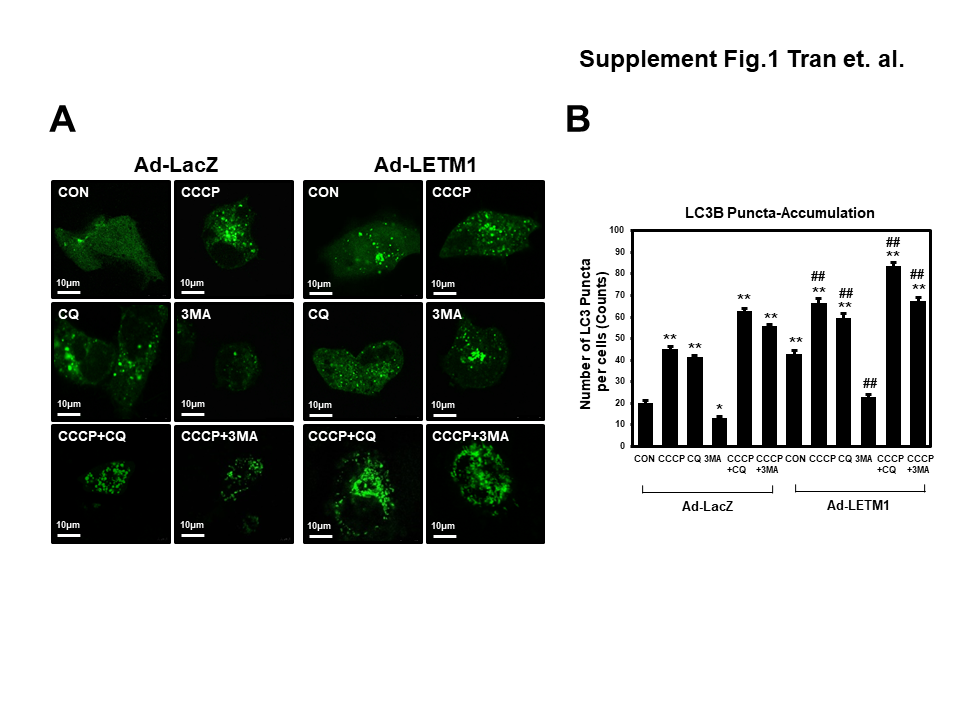

Supplement: Supplementary file 3 — Supple figure 1 [file 41419_2022_4993_MOESM3_ESM.png]

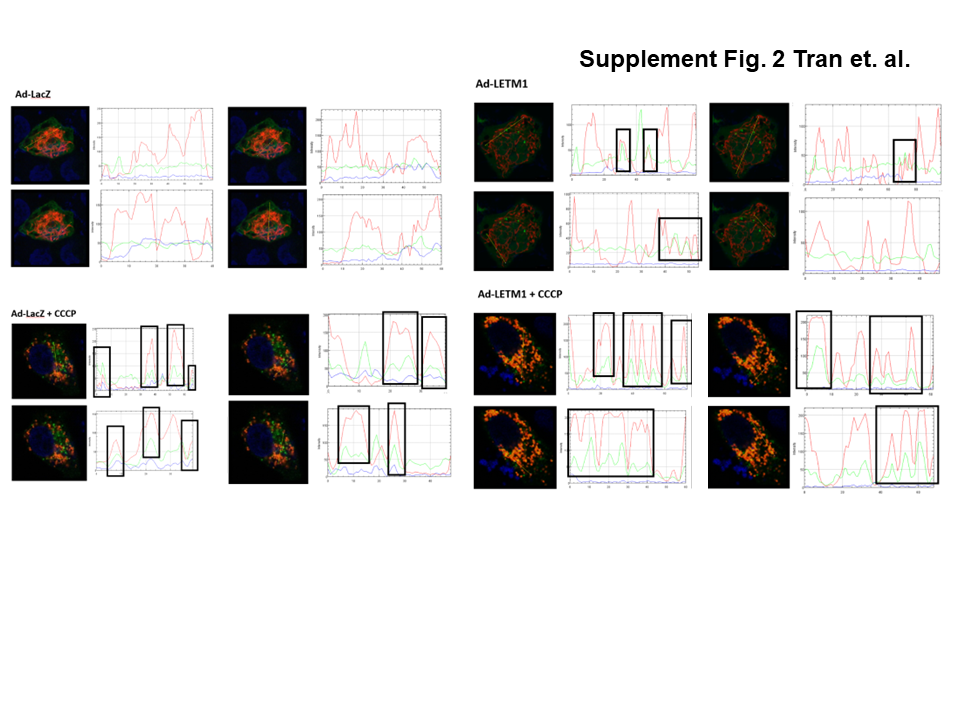

Supplement: Supplementary file 4 — Supple figure 2 [file 41419_2022_4993_MOESM4_ESM.png]

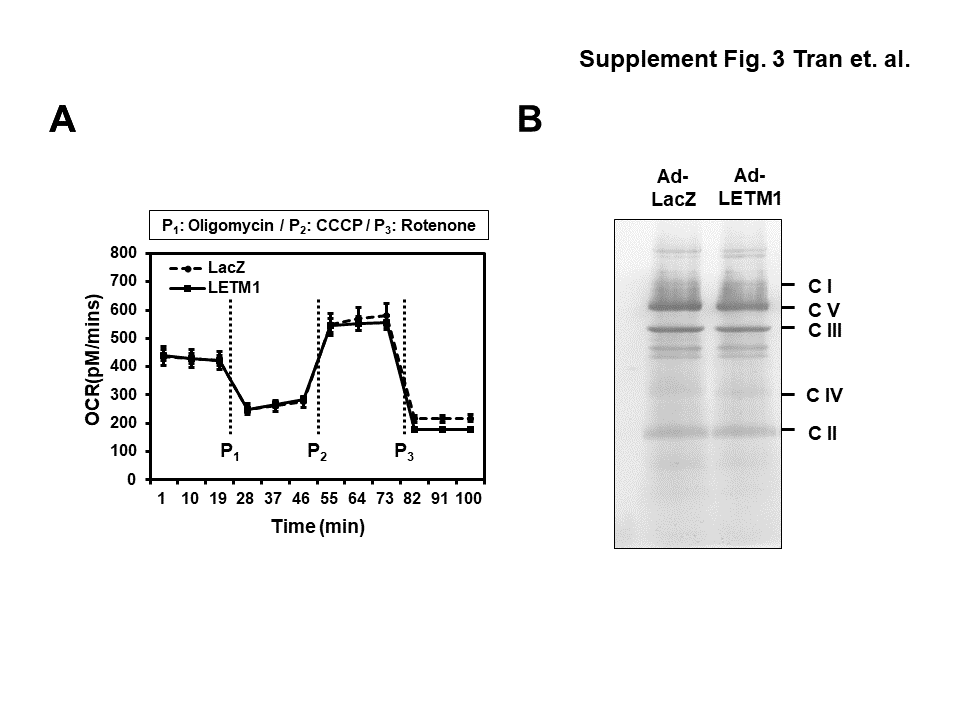

Supplement: Supplementary file 5 — Supple figure 3 [file 41419_2022_4993_MOESM5_ESM.png]
