# Supplementary material for: Emerging role of LETM1/GRP78 axis in lung cancer
Source: Cell Death Dis. 2022 Jun 10;13(6):543. doi: 10.1038/s41419-022-04993-5 (PMC9184611; doi:10.1038/s41419-022-04993-5)
Supplement: Supplementary file 6 — Dataset Western blot [file 41419_2022_4993_MOESM6_ESM.pdf]

Figure 1E anti-LETM1

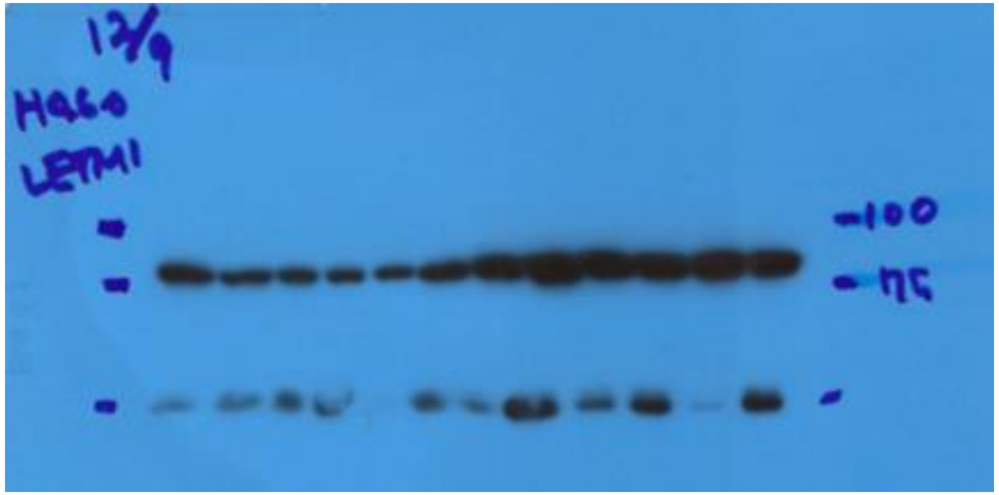

Figure 1E anti-LC3B

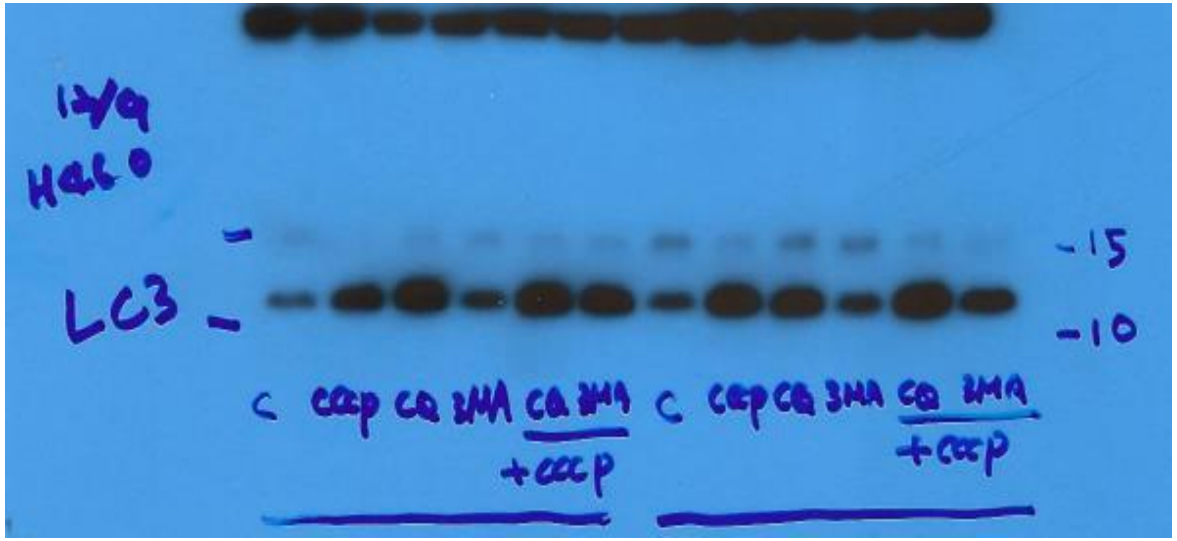

Figure 1E anti-p62

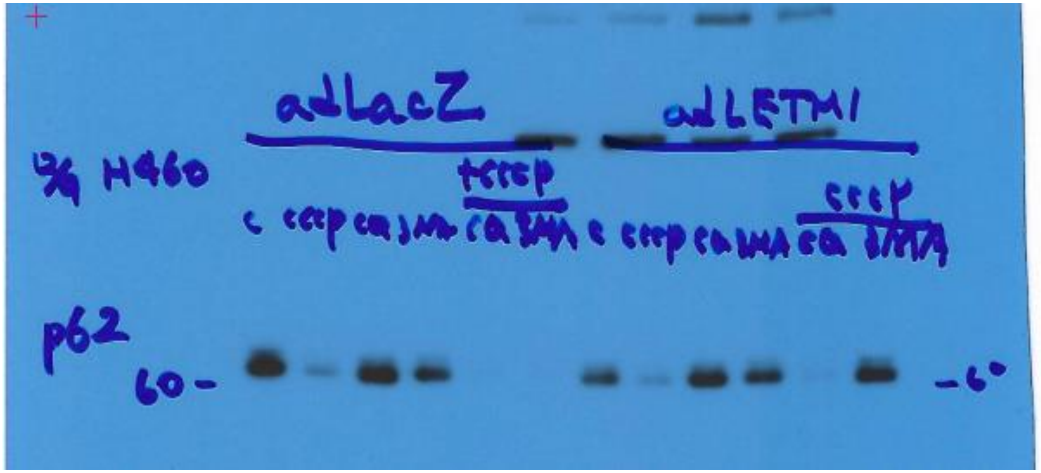

Figure 1E anti-beta-actin

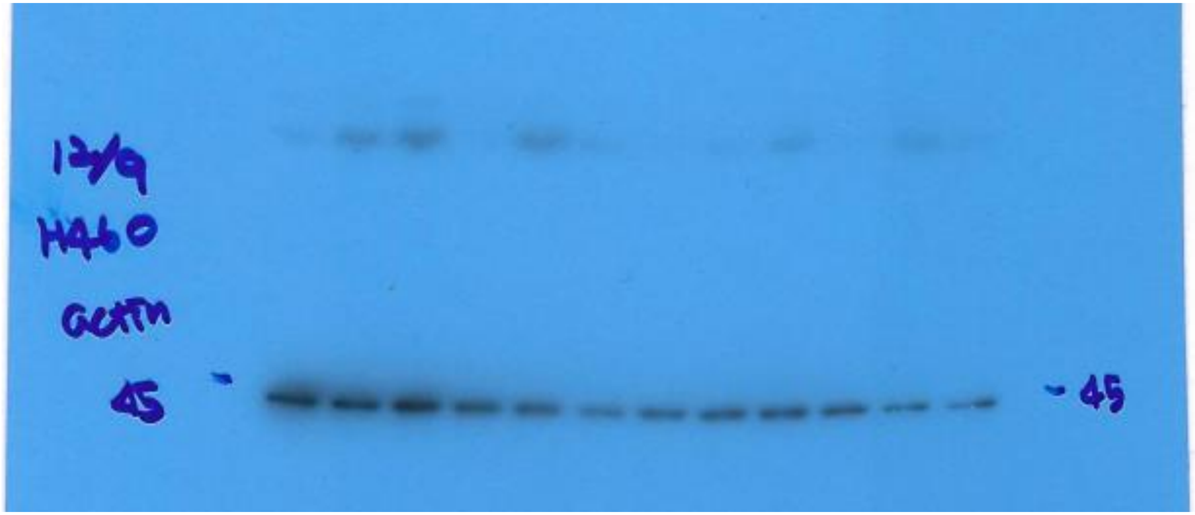

Figure 4E anti-GRP78

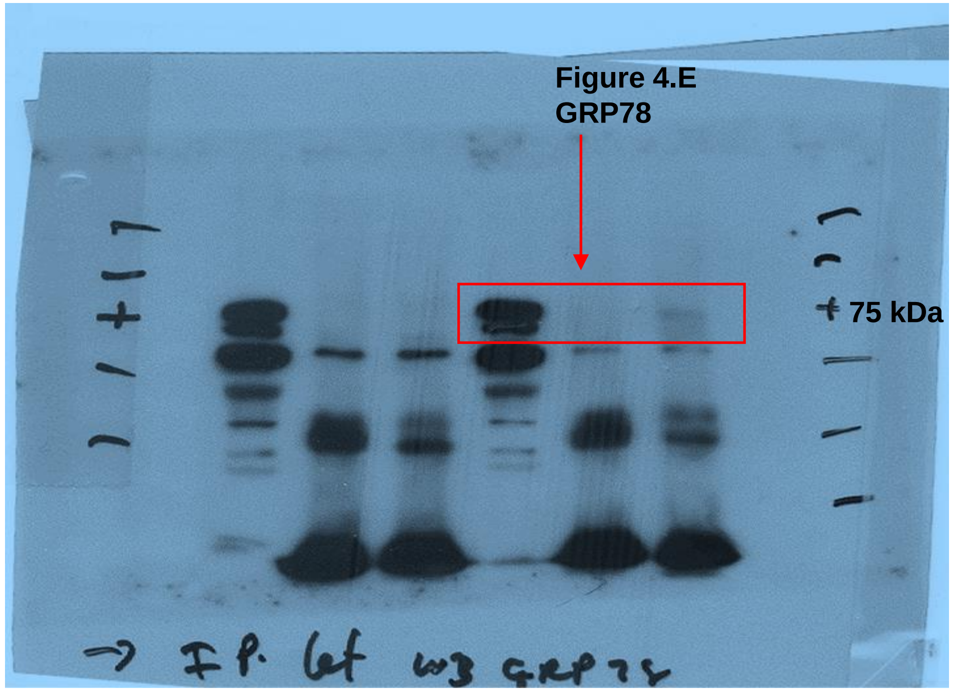

Figure 4E anti-LETM1

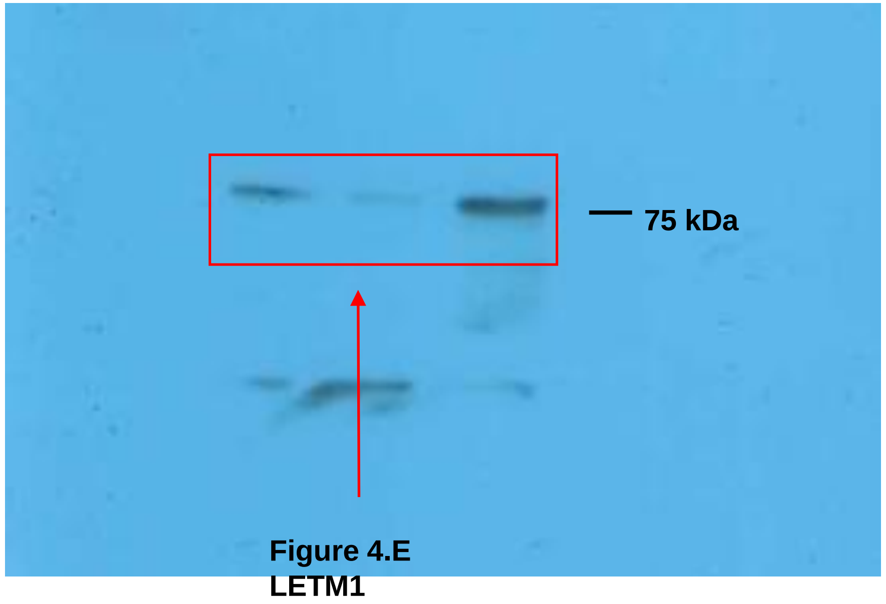

Figure 4F anti-GRP78

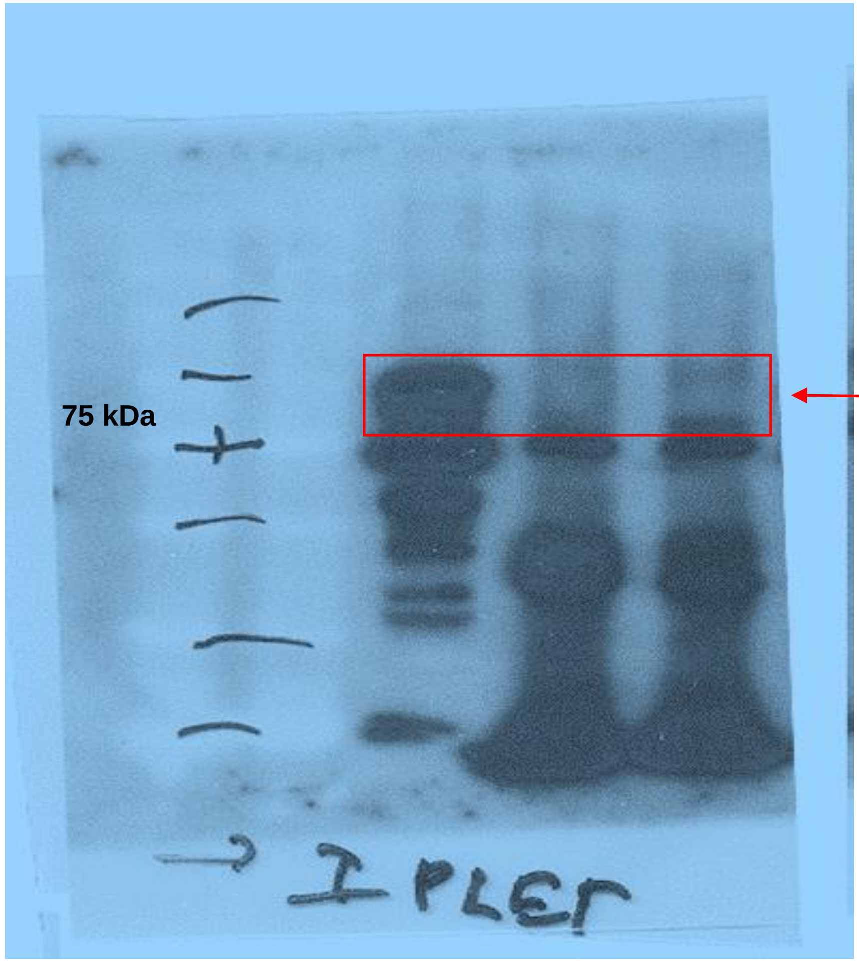

Figure 4F
GRP78

Figure 4F anti-LETM1

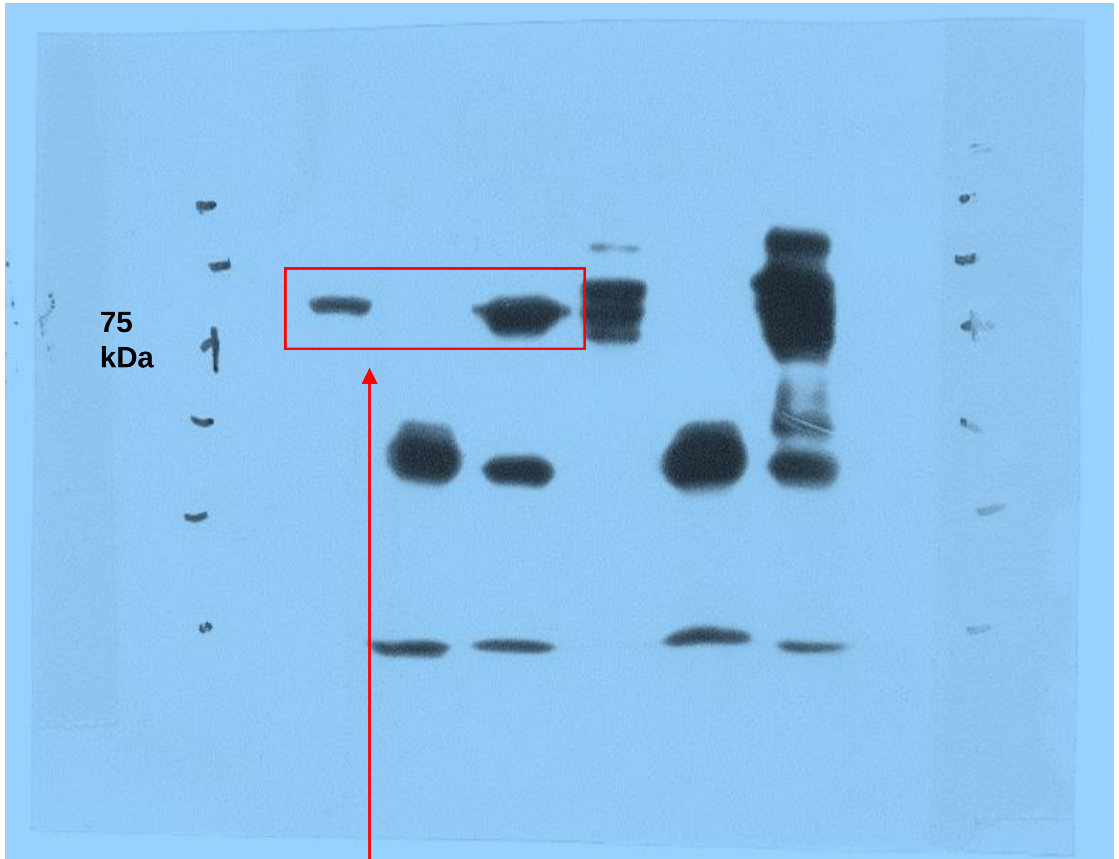

Figure 4F
LETM1

Figure 5B anti-GRP78

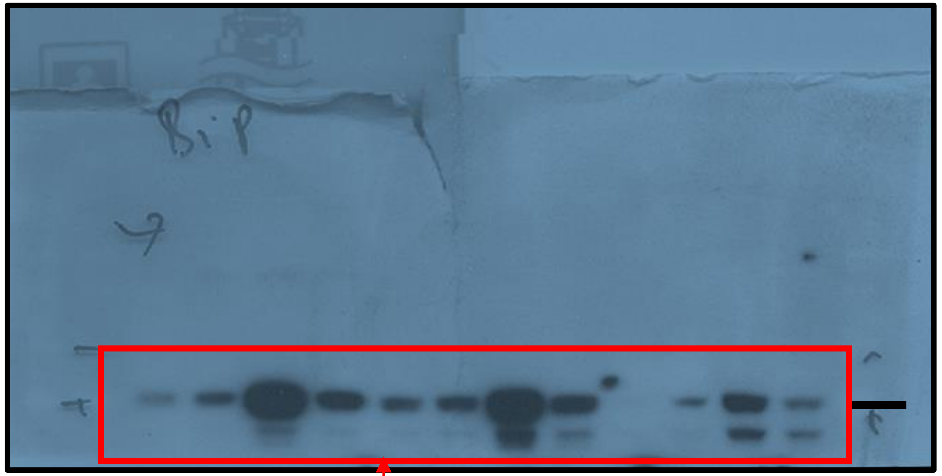

75 kDa

Figure 5B, GRP78

Figure 5B anti-TOM40

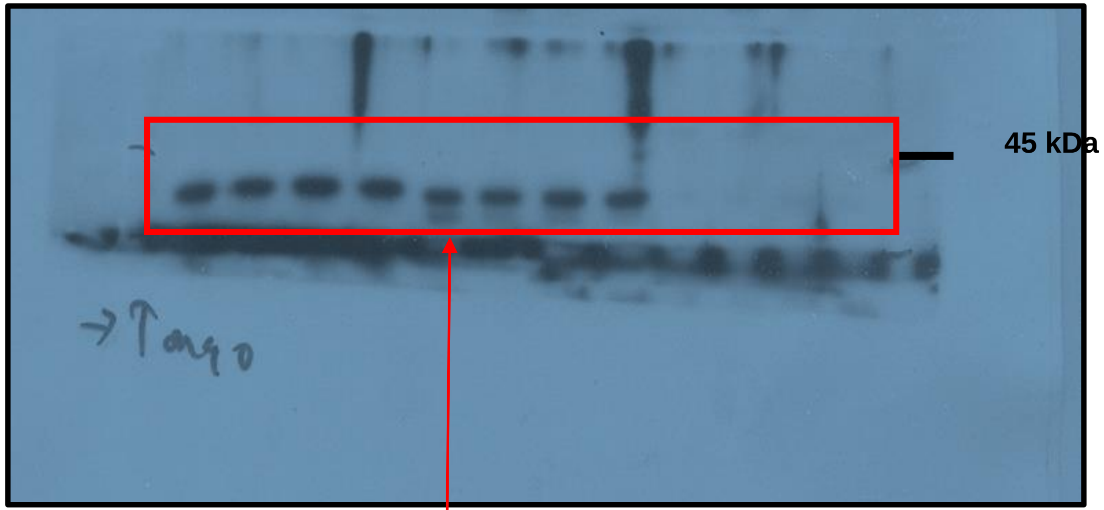

45 kDa

Figure 5B, TOM40

Figure 5B anti-LETM1

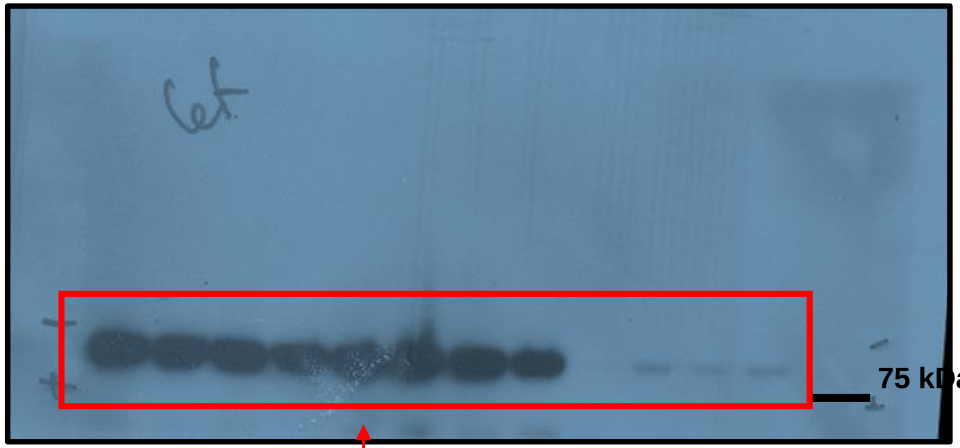

75 kDa

Figure 5B, LETM1

Figure 5B anti-beta-tubulin

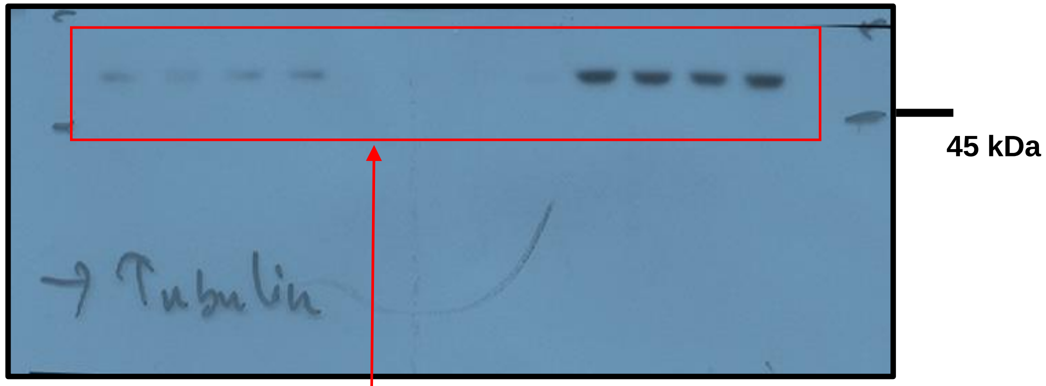

45 kDa

Figure 5B, Tubulin

Figure 5C anti-GRP78

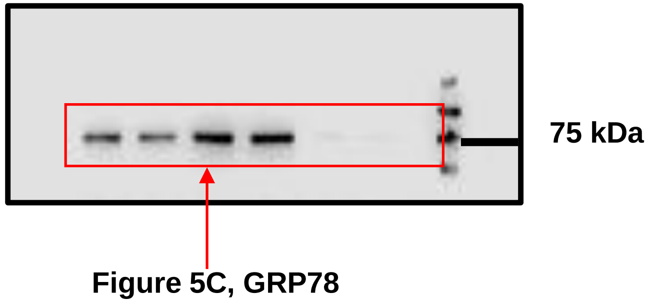

Figure 5C anti-TOM40

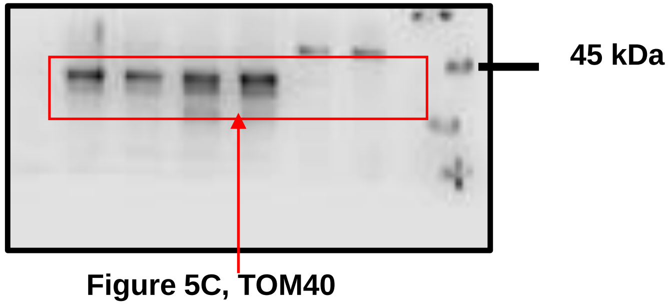

Figure 5C anti-LETM1

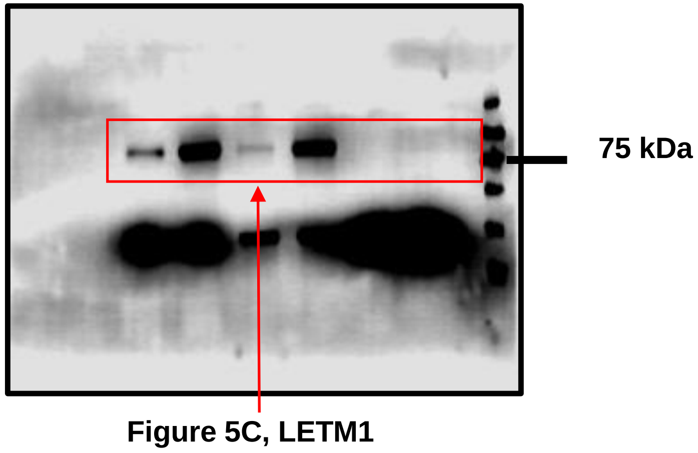

Figure 5C anti-beta-actin

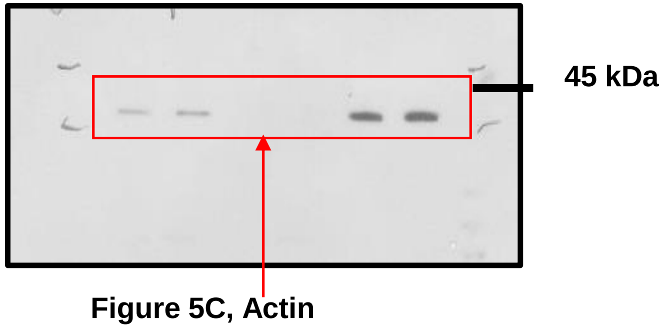

Figure 5E anti-LETM1

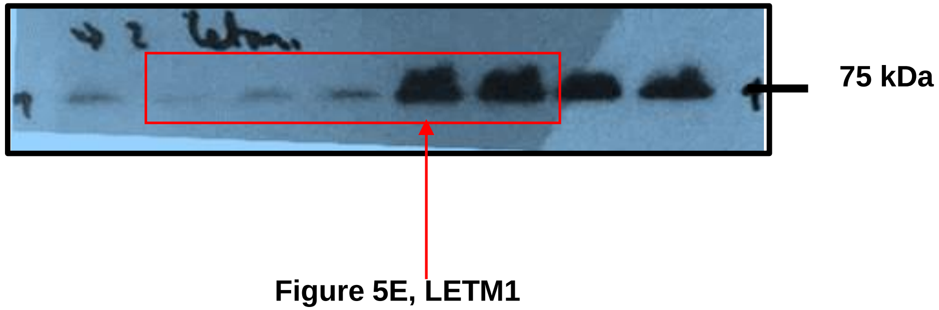

Figure 5E anti-beta-actin

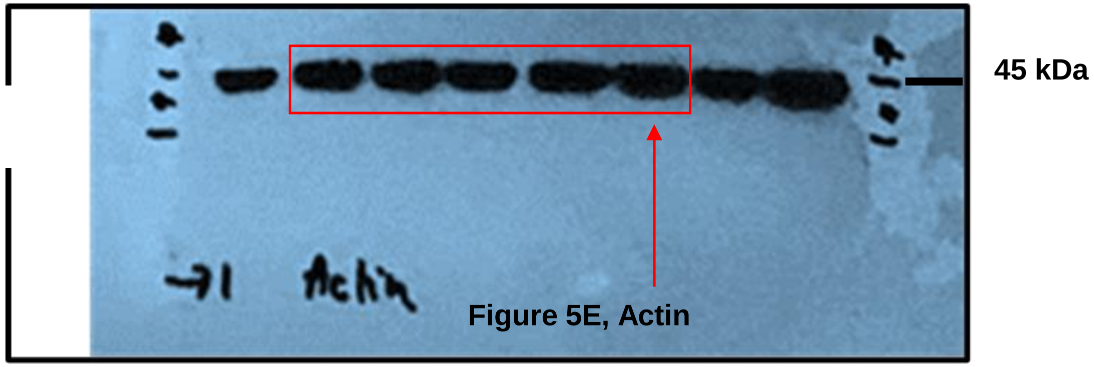

Figure 6C anti-GRP785 and anti-GRP78

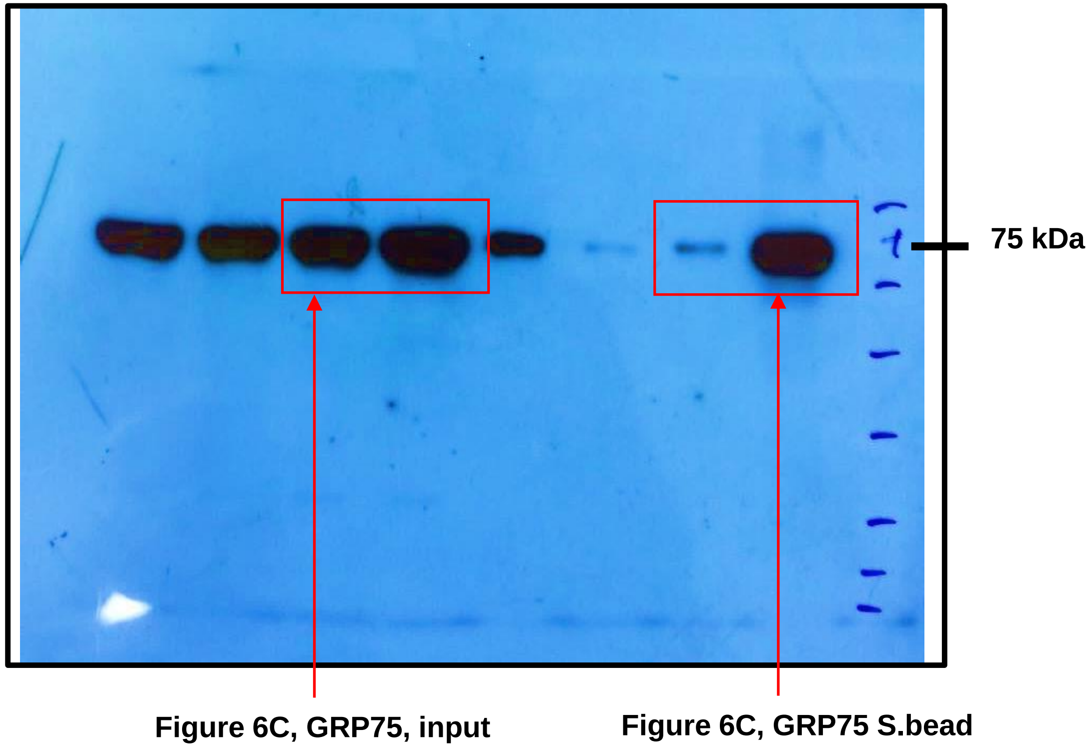

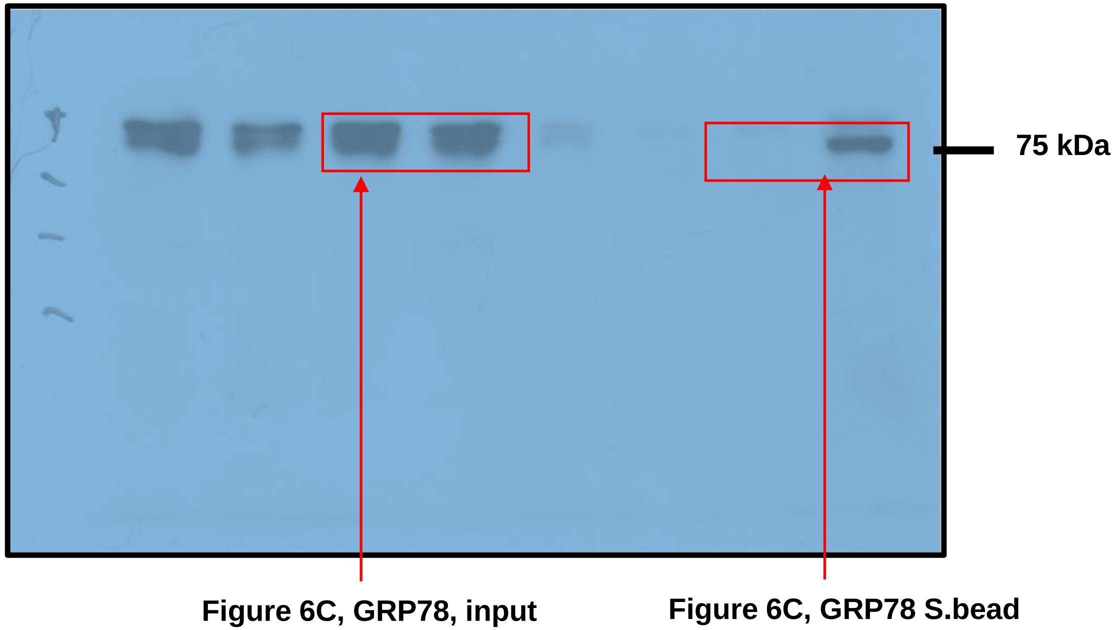

Figure 6D anti-GRP75

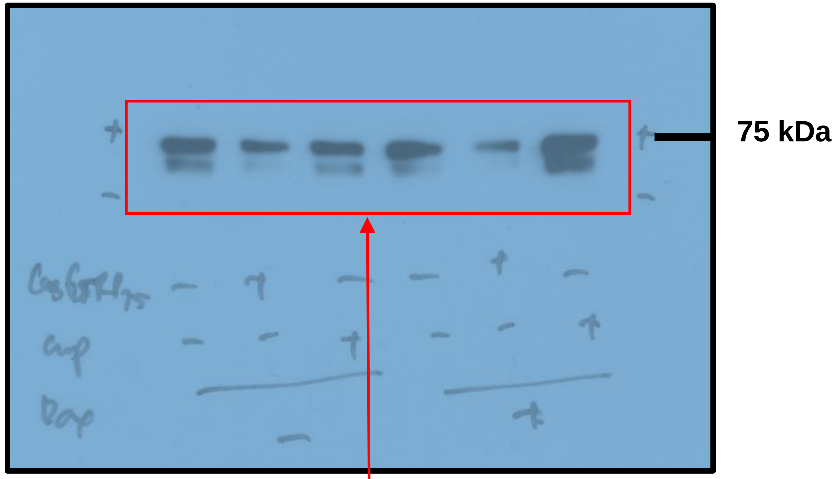

Figure 6D, GRP75

Figure 6D anti-LC3B

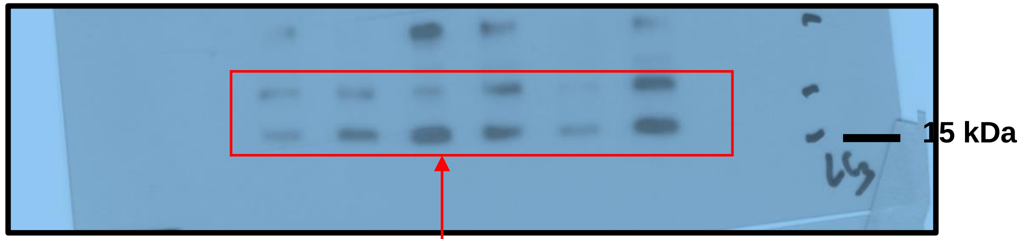

Figure 6D, LC3B

Figure 6D anti-LETM1

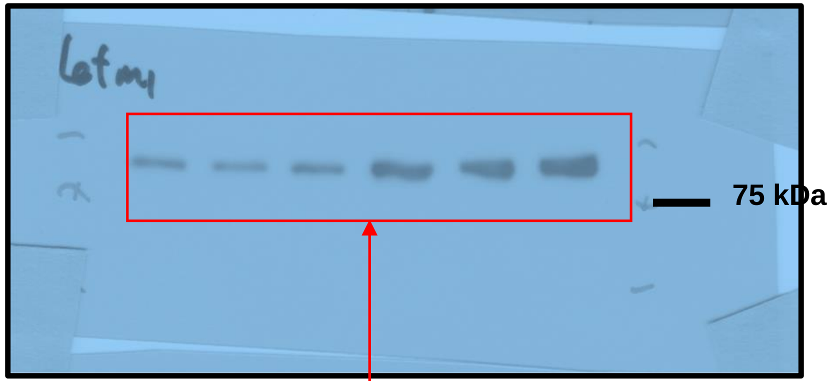

Figure 6D, LETM1

Figure 6D anti-beta-actin

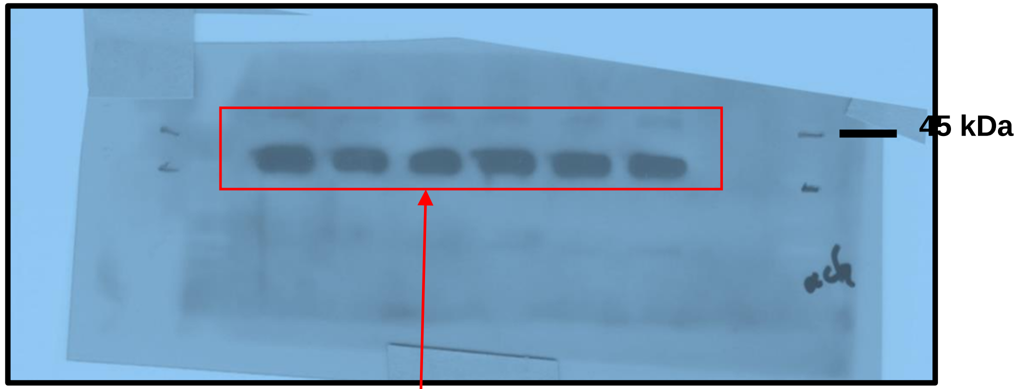

Figure 6D, Actin
